# Potential Antiviral Xanthones from a Coastal Saline Soil Fungus *Aspergillus iizukae*

**DOI:** 10.3390/md16110449

**Published:** 2018-11-15

**Authors:** Hui-Hui Kang, Huai-Bin Zhang, Mei-Jia Zhong, Li-Ying Ma, De-Sheng Liu, Wei-Zhong Liu, Hong Ren

**Affiliations:** 1 College of Pharmacy, Binzhou Medical University, Yantai 264003, China; kanghuihui_1993@126.com (H.-H.K.); zhanghuaibinhua@163.com (H.-B.Z.); 18660627015@163.com (M.-J.Z.); maliyingbz@163.com (L.-Y.M.); 2 Beijing Higher Institution Engineering Research Center of Food Additives and Ingredients, Beijing Key Laboratory of Flavor Chemistry, Beijing Laboratory for Food Quality and Safety, Beijing Technology and Business University, Beijing 100048, China; renhong@th.btbu.edu.cn

**Keywords:** *Aspergillus iizukae*, xanthones, antiviral activity, structure-activity relationship

## Abstract

Five new (**1**–**5**) and two known xanthones (**6** and **7**), one of the latter (**6**) obtained for the first time as a natural product, together with three known anthraquinones, questin, penipurdin A, and questinol, were isolated from the coastal saline soil-derived *Aspergillus iizukae* by application of an OSMAC (one strain many compounds) approach. Their structures were determined by interpretation of nuclear magnetic resonance (NMR) and high-resolution electrospray ionization mass spectroscopy (HRESIMS) data, as well as comparison of these data with those of related known compounds. Antiviral activity of xanthones **1**−**7** was evaluated through the cytopathic effect (CPE) inhibition assay, and compound **2** exhibited distinctly strong activity towards influenza virus (H1N1), herpes simplex virus types 1 (HSV-1) and 2 (HSV-2) with IC_50_ values of 44.6, 21.4, and 76.7 μM, respectively, which indicated that it was worth to further investigate it as a potential lead compound. The preliminary structure-activity relationship of the xanthones is discussed.

## 1. Introduction

*Aspergillus iizukae* is present in various environments, such as leaves of *Silybum marianum* [1], sponges [2], guts of a cricket species *Gryllus testaceus* [3], earthworm casts and different kinds of soil [4]. Recently, Özkaya et al found that the ethyl acetate extract of a sponge-associated *A. iizukae* showed inhibitory effects against the aquaculture pathogens *Lactococcus garvieae* and *Vagococcus salmoninarum* [2]. Additionally, a novel flavin adenine dinucleotide-dependent glucose dehydrogenase was separated from the culture of *A. iizukae*, which was capable of catalyzing the oxidation of glucose to glucono-δ-lactone [5]. Nevertheless, there are only a couple of reports on its metabolites. Up to now, four new and three known aromatic butenolides were isolated from the insect symbiont *A. iizukae* [3]. It was also reported that the endophytic *A. iizukae* from *S. marianum* could produce silybin A, silybin B and isosilybin A [1].

**Figure 1:**
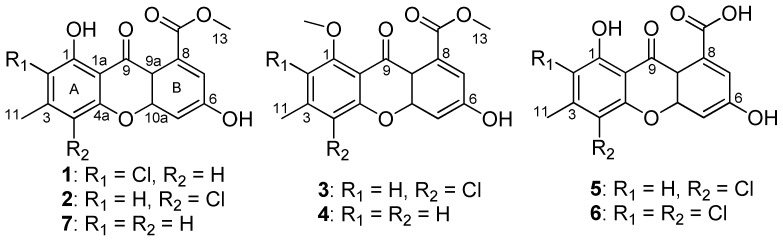
Structures of **1**–**7**.

In our continuing search for new biologically active metabolites from fungi [6,7], the prolific fungus *A. iizukae* was isolated from coastal saline soil in Kenli, China. Previously, two new diphenyl derivatives and nine known compounds were obtained from it in a shaken fermentation, and six of them contain chloride atoms, that demonstrating the strain was able to utilize chlorine in the biosynthesis of its metabolites [8]. In order to obtain more halogen-containing compounds, the working strain was fermented statically in liquid culture medium supplemented with sodium bromide applying an OSMAC strategy. Chemical exploration of the fungal extract led to the isolation of seven xanthones (**1**−**7**) (Figure 1), including five new xanthones (**1**−**5**), one new natural product (**6**) previously reported as a semisynthetic compound, and a known one (**7**), along with three known anthraquinones. Herein, the isolation and structure elucidation of the new metabolites, along with the antiviral activity of **1**−**7** are reported.

## 2. Results

**Figure 2:**
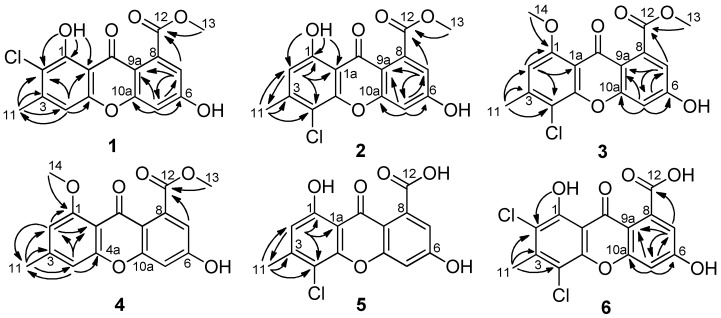
Selected key HMBC correlations of **1**–**6**.

Compound **1** was obtained as a yellow amorphous powder. Its molecular formula C_16_H_11_ClO_6_, was established by the high-resolution electrospray ionization mass spectroscopy (HRESIMS) *m*/*z* 335.0316 [M + H]^+^, revealing eleven degrees of unsaturation. The infrared (IR) spectrum revealed the presence of hydroxy (3219 cm^−1^), conjugated ketone carbonyl (1704 cm^−1^), conjugated ester carbonyl (1645 cm^−1^), and aromatic (1608, 1574, 1493 and 1437 cm^−1^) groups. The ultraviolet (UV) spectrum maxima at 358, 312, 290 and 237 nm showed typical absorptions of xanthones [9]. The ^1^H NMR spectrum (Table 1) presented two phenolic hydroxy groups at δ_H_ 13.00 (br s, 1-OH) and 11.63 (br s, 6-OH), an aromatic singlet at δ_H_ 7.12 (s, H-4), two doublets of *meta*-coupled aromatic protons at δ_H_ 6.96 (d, *J* = 2.1 Hz, H-5) and 6.89 (d, *J* = 2.1 Hz, H-7) , one methoxy group at δ_H_ 3.88 (s, Me-13), and one aromatic methyl group at δ_H_ 2.46 (s, Me-11). The ^13^C NMR data (Table 1) displayed sixteen carbon signals, including two conjugated carbonyl groups at δ_C_ 178.9 and 168.1, twelve aromatic carbon signals (three methines and four oxygenated quaternary carbons) in the region δ_C_ 103.3–164.4, one methoxy group at δ_C_ 52.7 and one methyl group at δ_C_ 20.8, which were clearly supported by the heteronuclear single quantum coherence (HSQC) data. The above information suggested **1** to be a chlorinated derivative of methyl-(l,6-dihydroxy-3-methylxanthone)-8-carboxylate (**7**) [10], which was also isolated from this culture. The positions of the carbomethoxy and hydroxy groups in ring B were confirmed by the correlations from H-7 to C-5, C-12 and C-9a, and from H-5 to C-6, C-7, C-9a and C-10a in the heteronuclear multiple bond correlation (HMBC) data (Figure 2). The chemical shift (δ_C_ 178.9) of the carbonyl (C-9) indicated the presence of a hydrogen-bonded hydroxyl group at C-1 [11,12]. Additional HMBC correlations from 1-OH to C-1, C-2 and C-1a, and from H_3_-11 to C-2, C-3 and C-4, placed the chlorine atom on C-2. Therefore, the structure of **1** was determined as methyl-(2-chloro-l,6-dihydroxy-3-methylxanthone)-8-carboxylate.

Compound **2** was isolated as a light-yellow powder. The HRESIMS data (*m*/*z* 335.0319 [M + H]^+^) gave the same molecular formula C_16_H_11_ClO_6_ as **1**. The IR and UV data of **2** resembled those of **1**, indicating that they have same scaffold. Detailed analysis and comparison of their ^1^H and ^13^C NMR data revealed a slight difference in the chemical shifts of ^1^H and ^13^C atoms in the rings A, especially those of C-1 and C-4a, while chemical shifts in the rings B were almost identical between **1** and **2**. The aforementioned data suggested that the chlorine atom was at C-4 in **2**, which was confirmed by the HMBC correlations (Figure 2) from H_3_-11 to C-2, C-3 and C-4, and from 1-OH to C-1, C-2 and C-1a. The other HMBC correlations shown in Figure 2 supported that the structure of **2** is methyl-(4-chloro-l,6-dihydroxy-3-methylxanthone)-8-carboxylate.

Compound **3**, a light-yellow solid, was isolated in small amount, and showed a protonated ion in its HRESIMS at *m*/*z* 349.0476 [M + H]^+^, indicating a molecular formula of C_17_H_13_ClO_6_ having one CH_2_ unit more than that of **2**. The ^1^H and ^13^C NMR data were very similar to those of **2**, except for the appearance of an additional methoxyl group and the disappearance of a hydroxyl group. Therefore, **3** was a methylated derivative of **2**. The lower resonance frequencies of C-9 (δ_C_ 172.9) suggested that the methoxyl was at C-1 [13]. This hypothesis was proved by the HMBC correlations (Figure 2) from H_3_-14 to C-1, and from H-2 to C-1, C-4 and C-1a. Based on these evidences, the structure of **3** was established as methyl-(4-chloro-6-hydroxy-1-methoxy-3-methylxanthone)-8-carboxylate.

Compound **4** was obtained as an orange powder. The molecular formula was determined as C_17_H_14_O_6_ from the HRESIMS *m*/*z* 313.0727 [M − H]^−^, showing the absence of chlorine atoms and one hydrogen atom more than that of **3**. In the ^1^H NMR spectrum, four broad singlets (δ_H_ 6.94, 6.87 6.82 and 6.73, 1H, s, each) were observed. Careful comparison of the NMR spectra data between **3** and **4** (Table 1 and Table 2) revealed that it was a dechlorinated derivative of **3**. This hypothesis was confirmed by analysis of the HMBC correlations (Figure 2). Therefore, the structure of **4** was elucidated as methyl-(6-hydroxy-1-methoxy-3-methylxanthone)-8-carboxylate.

Compound **5** was isolated as a pale-yellow powder and had the molecular formula C_15_H_9_ClO_6_ as determined by the HRESIMS *m*/*z* 319.0013 [M − H]^−^. Therefore, **5** has one CH_2_ unit less than **2**. In its IR spectrum the broad absorption at 3300−2600 cm^−1^, along with the absorption at 1694 cm^−1^, revealed the presence of a carboxylate functionality in **5**. Its NMR data were very similar to those of **2**, except for the absence of a methoxy group and the presence of a broad singlet of a hydroxy group in **5**. In the HMBC spectrum, the correlations from H-11 to C-2, C-3 and C-4, and from H-2 to C-1, C-4, C-11 and C-1a were observed, which unequivocally established the substitution of ring A. In spite of no HMBC correlations observed from H-6 and H-7 to any carbons, the ring B and the chemical shift assignment of corresponding carbons and protons could be achieved by comparison of its ^1^H and ^13^C data with those of **6** and calyxanthone [14]. Interestingly, in the ^13^C NMR spectrum, the resonance intensity of carbon atoms in ring B was much weaker than that of carbon atoms in ring A (Figure S30). Compound **5** was identified as 4-chloro-1,6-dihydroxy-3-methylxanthone-8-carboxylic acid.

Compound **6** was isolated as an orange-red powder and has low solubility in methanol. Its molecular formula C_15_H_8_Cl_2_O_6_ was determined on the basis of the HRESIMS *m*/*z* 352.9604 [M − H]^−^. The relative height of the typical isotopic ion peak (at *m*/*z* 354.9570 [M − H]^−^) was approximately two thirds of that of the quasi-molecular ion peak (Figure S40), suggesting the existence of two chlorine atoms. Its UV spectrum showed characteristic absorption bands of xanthones. In the ^1^H NMR spectrum, the *meta*-coupled aromatic protons at δ_H_ 6.95 (d, *J* = 2.2 Hz, H-5) and 6.86 (d, *J* = 2.1 Hz, H-7) of ring B were supported by the HMBC correlations (Figure 2) from H-5 to C-6, C-7, C-9a and C-10a, and from H-7 to C-5, C-12 and C-9a, which was confirmed by similar ^1^H and ^13^C chemical shifts values to those of calyxanthone [14]. The downfield shift of the carbonyl implied the hydrogen bonded phenolic hydroxyl at δ_H_ 13.22 (br s, 1-OH) attached to C-1. Additionally, the HMBC correlations from the aromatic methyl protons to C-2, C-3 and C-4, and from 1-OH to C-2, demonstrated that chlorine atoms were both at C-4 and C-2, respectively. Ultimately, the structure of **6** was established to be 2,4-dichloro-1,6-dihydroxy-3-methylxanthone-8-carboxylic acid. Compound **6** was previously described as a synthetic intermediate using only UV and IR data [15]. This is the first report of its isolation from a natural extract.

The known compounds were identified as methyl-(l,6-dihydroxy-3-methylxanthone)-8-carboxylate (**7**) [14], questin [8], penipurdin A [16], and questinol [17] by comparison of their NMR data with those reported in the literatures.

Xanthones **1**−**7** were screened for their antiviral activity against H1N1, HSV-1 and HSV-2 using the CPE inhibition assay (Table 3). Compounds **1**, **2** and **7** exhibited anti-H1N1 activity with IC_50_ values of 133.4, 54.6 and 140.4 μM, respectively, while the others were inactive (ribavirin was used as the positive control, IC_50_ 101.4 μM). Compounds **1**, **2** and **7** showed a strong anti-HSV-1 activity with IC_50_ values of 55.5, 21.4 and 75.7 μM, respectively, and the other compounds showed a moderate anti-HSV-1 activity compared with the positive control (acyclovir, IC_50_ 150.2 μM). Compounds **2** and **7** also possessed a strong anti-HSV-2 effect with IC_50_ values of 76.7 and 95.4 μM (acyclovir as the positive control, IC_50_ 128.6 μM), respectively.

## 3. Materials and Methods

### 3.1. General Experimental Procedures

Instrumentation used to acquire UV, IR, HRESIMS, optical rotation, and 1D and 2D NMR spectra and to perform column chromatography has been previously described [6,7,8] A TU-1091 spectrophotometer (Beijing Purkinje General Instrument Co., Ltd., Beijing, China) was used to measure the UV spectra in MeOH. An attenuated total reflection (ATR) method was employed to record the infrared spectra on a Nicolet 6700 spectrophotometer (Thermo Fisher Scientific, Madison, WI, USA). An Autopol V Plus digital polarimeter (Rudolph Research Analytical, Hackettstown, NJ, USA) was used to measure optical rotation. Detailed 1D and 2D NMR spectra were recorded on a Bruker AV-400 or Bruker AVIII 500 spectrometers (Bruker Biospin Group, Karlsruhe, Germany) with tetramethylsilane as an internal reference. A 1200RRLC-6520 Accurate-Mass Q-TOF LC/MS mass spectrometer (Agilent Technologies, Ltd., Palo Alto, CA, USA) was used to acquire HRESIMS spectra. HPLC purification was carried on a SHIMADZU LC-6AR (Shimadzu Corporation, Kyoto, Japan) Liquid Chromatograph equipped with an SPD-20A diode array detector, using an ODS column (HyperClone 5 μm ODS (C_18_), 120 Å, 250 mm × 10 mm, Phenomenex; Shim-pack GIS, 5 μm C_18_, 250 mm × 10 mm, Shimadzu. 4 mL/min).

### 3.2. Fungal Material

*Aspergillus iizukae* KL33 (GenBank accession numbers: HQ717800) was isolated from coastal saline soil in Kenli, Shandong Province of China, in August 2008. The strain was deposited at the Department of Chemistry, Binzhou Medical University, Yantai.

### 3.3. Fermentation and Extraction

*A. iizukae* KL33 was transferred aseptically to fresh PDA culture plates, and incubated at 28 °C for one week. Emerging fungal colonies were transferred into 500 mL Erlenmeyer flasks containing 180 mL of culture medium composed of glucose (20 g), maltose (10 g), mannitol (10 g), peptone (10 g), corn syrup (3 g), KH_2_PO_4_ (0.5 g), MgSO_4_·7H_2_O (0.3 g), sodium glutamate (10 g), sodium bromide (10 g), water (1 L, half seawater and half tap water) and statically fermented at room temperature for 6 weeks.

40 L of the fermentation broth was separated into mycelium and filtrate through cheesecloth. The filtrate was extracted three times with ethyl acetate. The mycelium was extracted with methanol for three times. The methanol solution was concentrated under reduced pressure to give an aqueous solution. The aqueous solution was extracted three more times with ethyl acetate. Both the ethyl acetate solutions were concentrated under reduced pressure to give a crude extract (65 g).

### 3.4. Purification

The crude extract (65 g) was fractionated into ten fractions (Frs 1−10) on a silica gel (200−300 mesh) column chromatography using a gradient of petroleum ether/chloroform (2:1, 1:1, 1:2, and 0:1, *v*/*v*), followed by chloroform/methanol (100:1, 50:1, 10:1, and 0:1, *v*/*v*). Fr. 2 was passed through an ODS column (25−40 μm, Merck, Darmstadt, Germany) using a sequential mixture of MeOH and H_2_O as eluent from 20% to 100% to obtain nine fractions (Frs 2.1−2.9). Similarly, Fr. 5 was fractionated into eight fractions (Frs 5.1−5.8). Fr. 2.5 and Fr. 2.3 were purified by semipreparative HPLC on an ODS column (shim-pack GIS) with MeOH/0.2% trifluoroacetic acid (TFA) aqueous solution (*v*/*v*) (4:1, *v*/*v*; 4 mL/min) as the mobile phase to yield **1** (20.3 mg, *t*_R_ 17.5 min) and **2** (18.6 mg, *t*_R_ 20.5 min), respectively. Fr. 4 was fractionated into three fractions (Frs 4.1−4.3) by an ODS column chromatograph (MeOH/H_2_O, 7:3, *v*/*v*). Fr. 4.1 was chromatographied on a silica gel column chromatography (CHCl_3_/MeOH, 50:1, *v*/*v*), and then purified by semipreparative HPLC on an ODS column (Phenomenex) eluted with MeOH/0.2% TFA aqueous solution (*v*/*v*) (70:30, *v*/*v*, 4 mL/min) to give **3** (10.3 mg, *t*_R_ 11.9 min) and **4** (7.3 mg, *t*_R_ 7.2 min). Fr. 6 was passed through ODS column (MeOH/H_2_O, 1:1, *v*/*v*), Sephadex LH-20 (MeOH), and semipreparative HPLC [shim-pack GIS, MeOH/0.2% TFA aqueous solution (*v*/*v*) (70:30, *v*/*v*; 4 mL/min)] successively to yield **5** (15.4 mg, *t*_R_ 22.8 min). Fr. 5.5, Fr. 2.4 and Fr. 5.4 were chromatographed on Sephadex LH-20 columns eluted with MeOH to obtain **6** (18.9 mg), **7** (17.5 mg), **9** (3.7 mg), respectively. Fr. 3 was purified by ODS (MeOH/H_2_O, 7:3, *v*/*v*) and Sephadex LH-20 columns (MeOH) to obtain **8** (17.3 mg). Fr. 5.3 was applied on Sephadex LH-20 (MeOH) column and further purified by semipreparative HPLC on an ODS column (Phenomenex) with MeOH/0.2% TFA aqueous solution (*v*/*v*) (55:45, *v*/*v*; 4 mL/min) as the eluting solvent to afford **10** (17.0 mg, *t*_R_ 12.1 min).

Compound **1**: a yellow amorphous powder; UV (MeOH) λ_max_ (log ε) 358 (3.92), 312 (4.09), 290 (3.97), 237 (4.46), 204 (4.29) nm; IR (ATR) ν_max_ 3219, 1704, 1645, 1608, 1574, 1493, 1437, 1414, 1381, 1273, 1231, 1176, 1146, 1024, 949, 848, 816, 775 cm^−1^; HRESIMS *m*/*z* 335.0316 [M + H]^+^ (calculated for C_16_H_12_ClO_6_, 335.0317). ^1^H and ^13^C NMR data: see Table 1.

Compound **2**: a light-yellow powder; UV (MeOH) λ_max_ (log ε): 360 (3.98), 307 (4.12), 272 (4.09), 253 (4.31), 235 (4.51), 204 (4.31) nm; IR (ATR) ν_max_ 3094, 2960, 1683, 1646, 1599, 1511, 1473, 1441, 1371, 1261, 1175, 1152, 907, 882, 811, 766 cm^−1^; HRESIMS *m*/*z* 335.0319 [M + H]^+^ (calculated for C_16_H_12_ClO_6_, 335.0317). ^1^H and ^13^C NMR data: see Table 1.

Compound **3**: a light-yellow solid; UV (MeOH) λ_max_ (log ε): 347 (3.59), 297 (3.74), 250 (3.97), 235 (4.12), 203 (4.12) nm; IR (ATR) ν_max_ 3203, 1740, 1560, 1584, 1480, 1436, 1325, 1225, 1151, 903, 886, 815, 770 cm^−1^; HRESIMS *m*/*z* 349.0476 [M + H]^+^ (calculated for C_17_H_14_ClO_6_, 349.0473). ^1^H and ^13^C NMR data: see Table 1.

Compound **4**: an orange powder; UV (MeOH) λ_max_ (log ε): 339 (3.38), 300 (3.53), 290 (3.55), 246 (3.78), 234 (3.93), 203 (3.98) nm; IR (ATR) ν_max_ 3392, 1740, 1678, 1620, 1607, 1437, 1197, 1135, 842, 822, 802, 724 cm^−1^; HRESIMS *m*/*z* 313.0727 [M − H]^−^ (calculated for C_17_H_1__3_O_6_, 313.0718). ^1^H and ^13^C NMR data: see Table 2.

Compound **5**: a pale-yellow powder; UV (MeOH) λ_max_ (log ε): 355 (3.81), 306 (4.21), 250 (4.40), 237 (4.53), 204 (4.37) nm; IR (ATR) ν_max_ 3101, 1694, 1644, 1607, 1558, 1506, 1475, 1239, 1149, 896, 821, 717 cm^−1^; HRESIMS *m*/*z* 319.0013 [M − H]^−^ (calculated for C_15_H_8_ClO_6_, 319.0015). ^1^H and ^13^C NMR data: see Table 2.

Compound **6**: an orange-red powder; UV (MeOH) λ_max_ (log ε): 361 (3.96), 312 (4.41), 240 (4.73), 203 (4.67) nm; IR (ATR) ν_max_ 3081, 1691, 1610, 1575, 1498, 1432, 1218, 1156, 913, 883, 828, 778 cm^−1^. HRESIMS *m*/*z* 352.9604 [M − H]^−^ (calculated for C_15_H_7_Cl_2_O_6_, 352.9625). ^1^H and ^13^C NMR data: see Table 2.

### 3.5. Antiviral Activity

The antiviral activity against influenza A virus (H1N1) was carried out by CPE inhibition assay as previously reported [18,19]. First, confluent MDCK cell monolayers and influenza virus (A/Puerto Rico/8/34 (H1N1), PR/8) were incubated together at 37 °C for 1 h. Then, the cells were treated with different test compounds after removing the virus dilution. After incubating at 37 °C for 48 h, the cells were fixed with 4% formaldehyde of 100 μL for 20 min at room temperature. Later on, the cells were stained with 0.1% crystal violet for 30 min after removal of the formaldehyde. Finally, the plates were washed and dried, followed by the measurement of the intensity of crystal violet staining for each well at 570 nm in a microplate reader (Bio-Rad, USA). Ribavirin was used as the positive control.

The anti-herpes simplex virus types 1 (HSV-1) and 2 (HSV-2) activity of **1**−**7** on Vero cells were conducted in the same way as described above [20], and acyclovir (ACV) was applied as the positive control.

## 4. Conclusions

In summary, the culture based on OSMAC strategy of the fungus *A. iizukae* yielded seven xanthones, including five new and one isolated for the first time as a natural product. Among them, five metabolites contain chlorine, and their structures were different from those previously reported from *A. iizukae*. From this study, it is clear that the OSMAC strategy is still a powerful tool in producing new metabolites from microorganisms. Compound **2** exhibited a strong antiviral activity against H1N1, HSV-1 and HSV-2 with IC_50_ values of 44.6, 21.4, and 76.7 μM, respectively, compared with the positive controls.

The results of antiviral activity of **1**−**7** indicated that the hydroxy group at C-1 and the methyl carboxylate group at C-8 essentially contributed to the anti-H1N1, anti-HSV-1 and anti-HSV-2 activities, and the position of the chlorine atom in ring A would affect the antiviral activities. Additionally, it seemed that methylation of the hydroxy group at C-1 or replacement of methyl carboxylate at C-8 by carboxylic acid, to a large extent, lower the antiviral effect.

Xathones have attracted considerable interest for their promising biological activities and the interesting structural scaffold, which could be modified by various substituents [21,22]. Our finding suggests that **2** might be a potential anti-H1N1 lead candidate, worthy of a further pharmacological exploration.

## Figures and Tables

**Table 1:** ^1^H and ^13^C NMR data for **1**–**3** (DMSO-*d*_6_).

Position	1 ^a^		2 ^a^		3 ^b^	
	δ_C_, type	δ_H_ (*J* in Hz)	δ_C_, type	δ_H_ (*J* in Hz)	δ_C_, type	δ_H_ (*J* in Hz)
1	155.8, C		158.6, C		158.4, C	
2	113.8, C		112.3, CH	6.83, s	109.3, CH	7.05, s
3	145.7, C		145.7, C		144.2, C	
4	108.7, CH	7.12, s	109.7, C		111.0, C	
5	103.3, CH	6.96, d (2.1)	103.3, CH	6.97, brs	103.3, CH	6.91, d (2.2)
6	164.4, C		164.5, C		163.3, C	
7	113.1, CH	6.89, d (2.1)	113.4, CH	6.89, brs	113.3, CH	6.78, d (2.2)
8	135.0, C		135.0, C		135.8, C	
9	178.9, C		178.9, C		172.9, C	
11	20.8, CH_3_	2.46, s	20.6, CH_3_	2.43, s	21.2, CH_3_	2.49, s
12	168.1, C		168.1, C		169.3, C	
13	52.7, CH_3_	3.88, s	52.7, CH_3_	3.88, s	52.9, CH_3_	3.84, s
14					56.8, CH_3_	3.88, s
1a	106.6, C		107.0, C		112.5, C	
4a	153.1, C		150.4, C		152.4, C	
9a	108.7, C		108.7, C		112.2, C	
10a	157.7, C		157.4, C		156.3, C	
HO-6		11.63, brs				11.25, brs
HO-1		13.00, s		12.29, brs		

^a 1^H (400 MHz) and ^13^C (100 MHz); ^b 1^H (500 MHz) and ^13^C (125 MHz).

**Table 2:** ^1^H and ^13^C NMR data for **4**–**6** (^1^H 400 MHz and ^13^C 100 MHz in DMSO-*d*_6_).

Position	4		5		6	
	δ_C_, type	δ_H_ (*J* in Hz)	δ_C_, type	δ_H_ (*J* in Hz)	δ_C_, type	δ_H_ (*J* in Hz)
1	159.8, C		158.7, C		154.6, C	
2	107.7, CH	6.82, s	112.3, CH	6.85 s	114.4, C	
3	146.5, C		145.6, C		142.6, C	
4	109.4, CH	6.94, s	109.6, C		110.6, C	
5	102.8, CH	6.87, s	102.8, CH	6.93, s	102.7, CH	6.95, d (2.2)
6	162.5, C		164.6, C		164.9, C	
7	112.2, CH	6.73, s	112.3, CH	6.85 s	113.1, CH	6.86, d (2.1)
8	135.4, C		137.4		137.2, C	
9	172.7, C		179.0, C		178.7, C	
11	21.8, CH_3_	2.42, s	20.6, CH_3_	2.44, s		
12	169.1, C		168.9, C		168.8, C	
13	52.4, CH_3_	3.84, s			18.5, CH_3_	2.56, s
14	56.1, CH_3_	3.87, s				
1a	109.2, C		107.0, C		107.1, C	
4a	156.9, C		150.4, C		149.0, C	
9a	112.2, C		108.3, C		108.0, C	
10a	156.1, C		157.5, C		157.5, C	
HO-6				12.44, s		
HO-1				12.44, s		13.22, brs

**Table 3:** Antiviral activity of xanthones **1**–**7** against H1N1, HSV-1 and HSV-2.

Compounds	IC_50_ (μM)							Acyclovir ^c^	Ribavirin ^c^
	1	2	3	4	5	6	7		
H1N1 ^a^	133.4	44.6	>200	>200	>200	>200	140.4		101.4
HSV-1 ^b^	55.5	21.4	139.4	157.7	183.3	144.4	75.7	150.2	
HSV-2 ^b^	175.5	76.7	>200	163.3	>200	>200	95.4	128.6	

^a^ Tested on MDCK cells; ^b^ Tested on Vero cells; ^c^ Positive control.

## Supplementary Materials

The following are available online at http://www.mdpi.com/1660-3397/16/11/449/s1, Figure S1: ^1^H NMR spectrum (400 MHz) of compound **1** in DMSO-*d*_6_, Figure S2: ^13^C NMR spectrum (100 MHz) of compound **1** in DMSO-*d*_6_, Figure S3: HSQC spectrum of compound **1** in DMSO-*d*_6_, Figure S4: HMBC spectrum of compound **1** in DMSO-*d*_6_, Figure S5: HRESIMS spectrum of compound **1**, Figure S6: IR spectrum of compound **1**, Figure S7: UV spectrum of compound **1** in MeOH, Figure S8: ^1^H NMR spectrum (400 MHz) of compound **2** in DMSO-*d*_6_, Figure S9: ^13^C NMR spectrum (100 MHz) of compound **2** in DMSO-*d*_6_, Figure S10: HSQC spectrum of compound **2** in DMSO-*d*_6_, Figure S11: HMBC spectrum of compound **2** in DMSO-*d*_6_, Figure S12: HRESIMS spectrum of compound **2**, Figure S13: IR spectrum of compound **2**, Figure S14: UV spectrum of compound **2** in MeOH, Figure S15: ^1^H NMR spectrum (500 MHz) of compound **3** in DMSO-*d*_6_, Figure S16: ^13^C NMR spectrum (125 MHz) of compound **3** in DMSO-*d*_6_, Figure S17: HSQC spectrum of compound **3** in DMSO-*d*_6_, Figure S18: HMBC spectrum of compound **3** in DMSO-*d*_6_, Figure S19: HRESIMS spectrum of compound **3**, Figure S20: IR spectrum of compound **3**, Figure S21: UV spectrum of compound **3** in MeOH, Figure S22: ^1^H NMR spectrum (400 MHz) of compound **4** in DMSO-*d*_6_, Figure S23: ^13^C NMR spectrum (100 MHz) of compound **4** in DMSO-*d*_6_, Figure S24: HSQC spectrum of compound **4** in DMSO-*d*_6_, Figure S25. HMBC spectrum of compound **4** in DMSO-*d*_6_, Figure S26: HRESIMS spectrum of compound **4**, Figure S27: IR spectrum of compound **4**, Figure S28: UV spectrum of compound **4** in MeOH, Figure S29: ^1^H NMR spectrum (400 MHz) of compound **5** in DMSO-*d*_6_, Figure S30: ^13^C NMR spectrum (100 MHz) of compound **5** in DMSO-*d*_6_, Figure S31: HSQC spectrum of compound **5** in DMSO-*d*_6_, Figure S32: HMBC spectrum of compound **5** in DMSO-*d*_6_, Figure S33: HRESIMS spectrum of compound **5**, Figure S34: IR spectrum of compound **5**, Figure S35: UV spectrum of compound **5** in MeOH, Figure S36: ^1^H NMR spectrum (400 MHz) of compound **6** in DMSO-*d*_6_, Figure S37: ^13^C NMR spectrum (100 MHz) of compound **6** in DMSO-*d*_6_, Figure S38: HSQC spectrum of compound **6** in DMSO-*d*_6_, Figure S39: HMBC spectrum of compound **6** in DMSO-*d*_6_, Figure S40: HRESIMS spectrum of compound **6**, Figure S41: IR spectrum of compound **6**, Figure S42: UV spectrum of compound **6** in MeOH.
