# Supplementary material for: Potential Antiviral Xanthones from a Coastal Saline Soil Fungus Aspergillus iizukae
Source: Mar Drugs. 2018 Nov 15;16(11):449. doi: 10.3390/md16110449 (PMC6265927; doi:10.3390/md16110449)
Supplement: Supplementary file 1 [file marinedrugs-16-00449-s001.pdf]

Potential Antiviral Xanthones from a Coastal Saline Soil Fungus *Aspergillus iizukae*

Hui-Hui Kang [1], Huai-Bin Zhang [1], Mei-Jia Zhong [1], Li-Ying Ma [1], De-Sheng Liu [1,*], Wei-Zhong Liu [1,*] and Hong Ren [2]

Figure S1. ^{1}H NMR spectrum (400 MHz) of compound **1** in DMSO-d_6 .... S3
Figure S2. ^{13}C NMR spectrum (100 MHz) of compound **1** in DMSO-d_6 .... S3
Figure S3. HSQC spectrum of compound **1** in DMSO-d_6 .... S4
Figure S4. HMBC spectrum of compound **1** in DMSO-d_6 .... S4
Figure S5. HRESIMS spectrum of compound **1** S5
Figure S6. IR spectrum of compound **1** S5
Figure S7. UV spectrum of compound **1** in MeOH S6
Figure S8. ^{1}H NMR spectrum (400 MHz) of compound **2** in DMSO-d_6 .... S6
Figure S9. ^{13}C NMR spectrum (100 MHz) of compound **2** in DMSO-d_6 .... S7
Figure S10. HSQC spectrum of compound **2** in DMSO-d_6 .... S7
Figure S11. HMBC spectrum of compound **2** in DMSO-d_6 .... S8
Figure S12. HRESIMS spectrum of compound **2** S8
Figure S13. IR spectrum of compound **2** S9
Figure S14. UV spectrum of compound **2** in MeOH S9
Figure S15. ^{1}H NMR spectrum (500 MHz) of compound **3** in DMSO-d_6 .... S10
Figure S16. ^{13}C NMR spectrum (125 MHz) of compound **3** in DMSO-d_6 .... S10
Figure S17. HSQC spectrum of compound **3** in DMSO-d_6 .... S11
Figure S18. HMBC spectrum of compound **3** in DMSO-d_6 .... S11
Figure S19. HRESIMS spectrum of compound **3** S12
Figure S20. IR spectrum of compound **3** S12
Figure S21. UV spectrum of compound **3** in MeOH S13
Figure S22. ^{1}H NMR spectrum (400 MHz) of compound **4** in DMSO-d_6 .... S13
Figure S23. ^{13}C NMR spectrum (100 MHz) of compound **4** in DMSO-d_6 .... S14
Figure S24. HSQC spectrum of compound **4** in DMSO-d_6 .... S14
Figure S25. HMBC spectrum of compound **4** in DMSO-d_6 .... S15
Figure S26. HRESIMS spectrum of compound **4** S15
Figure S27. IR spectrum of compound **4** S16
Figure S28. UV spectrum of compound **4** in MeOH S16

Figure S29. ^{1}H NMR spectrum (400 MHz) of compound **5** in DMSO-d_6 .... S17

Figure S30. ^{13}C NMR spectrum (100 MHz) of compound **5** in DMSO-d_6 .... S17

Figure S31. HSQC spectrum of compound **5** in DMSO-d_6 .... S18

Figure S32. HMBC spectrum of compound **5** in DMSO-d_6 .... S18

Figure S33. HRESIMS spectrum of compound **5** S19

Figure S34. IR spectrum of compound **5** S20

Figure S35. UV spectrum of compound **5** in MeOH S20

Figure S36. ^{1}H NMR spectrum (400 MHz) of compound **6** in DMSO-d_6 .... S20

Figure S37. ^{13}C NMR spectrum (100 MHz) of compound **6** in DMSO-d_6 .... S21

Figure S38. HSQC spectrum of compound **6** in DMSO-d_6 .... S21

Figure S39. HMBC spectrum of compound **6** in DMSO-d_6 .... S22

Figure S40. HRESIMS spectrum of compound **6** S22

Figure S41. IR spectrum of compound **6** S23

Figure S42. UV spectrum of compound **6** in MeOH S23

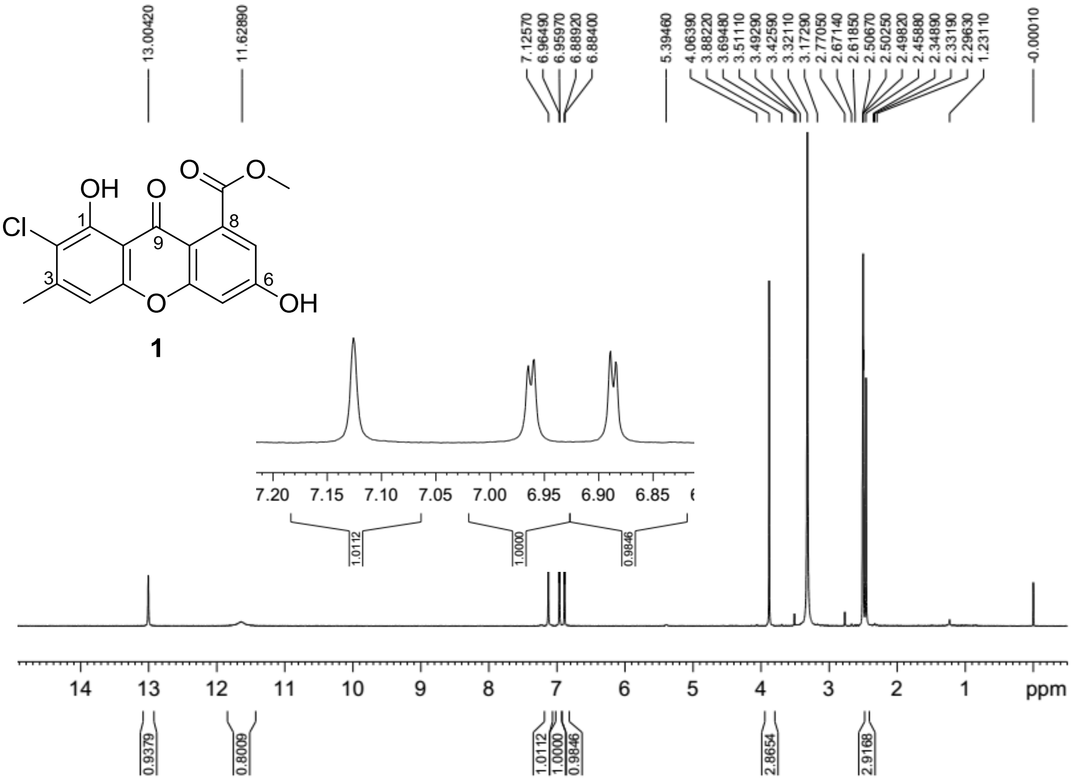

Figure S1. ^{1}H NMR spectrum (400 MHz) of compound **1** in DMSO-d_6

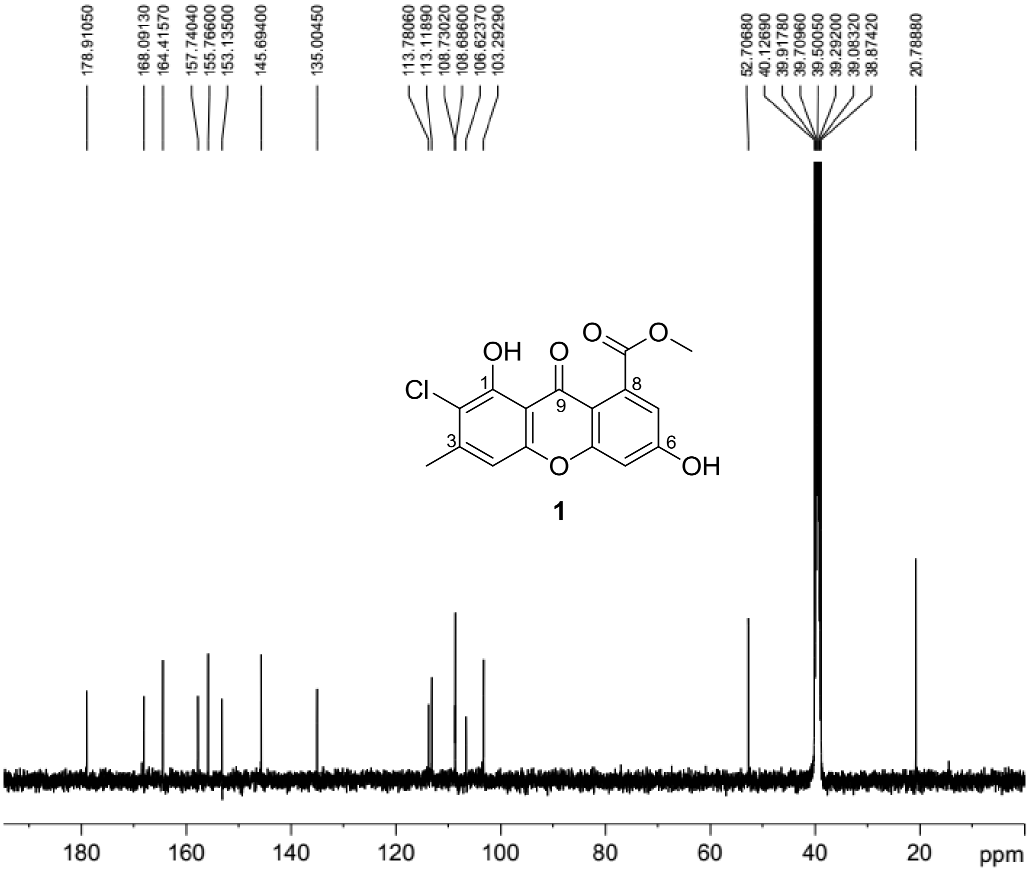

Figure S2. ^{13}C NMR spectrum (100 MHz) of compound **1** in DMSO-d_6

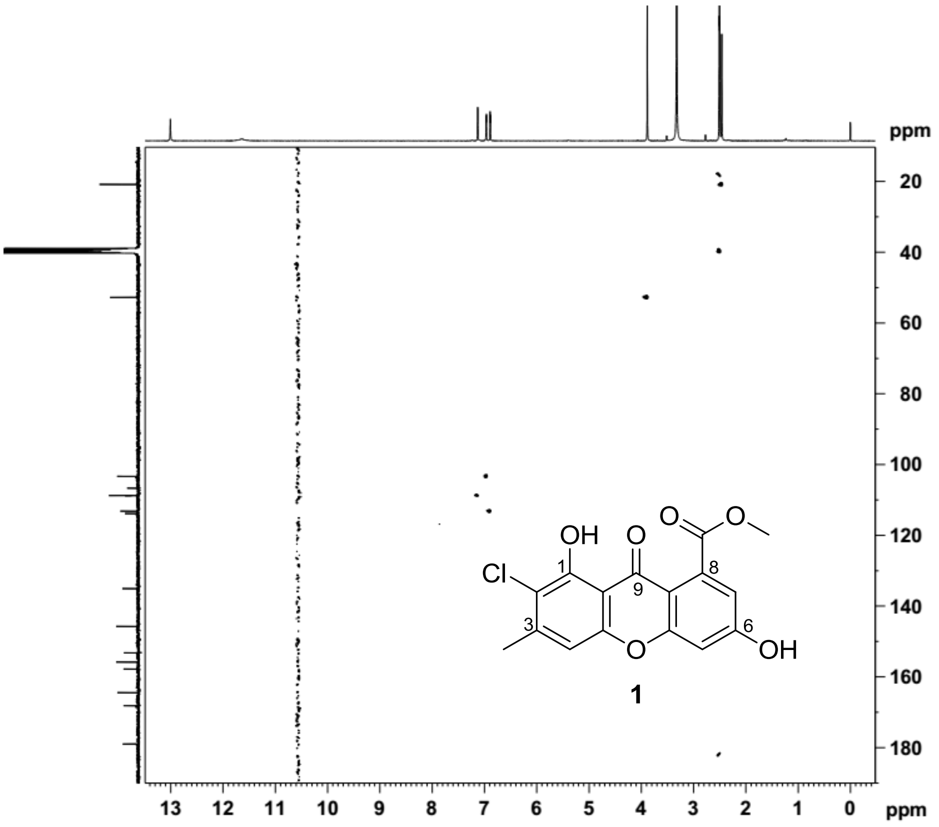

Figure S3. HSQC spectrum of compound **1** in DMSO-d_6

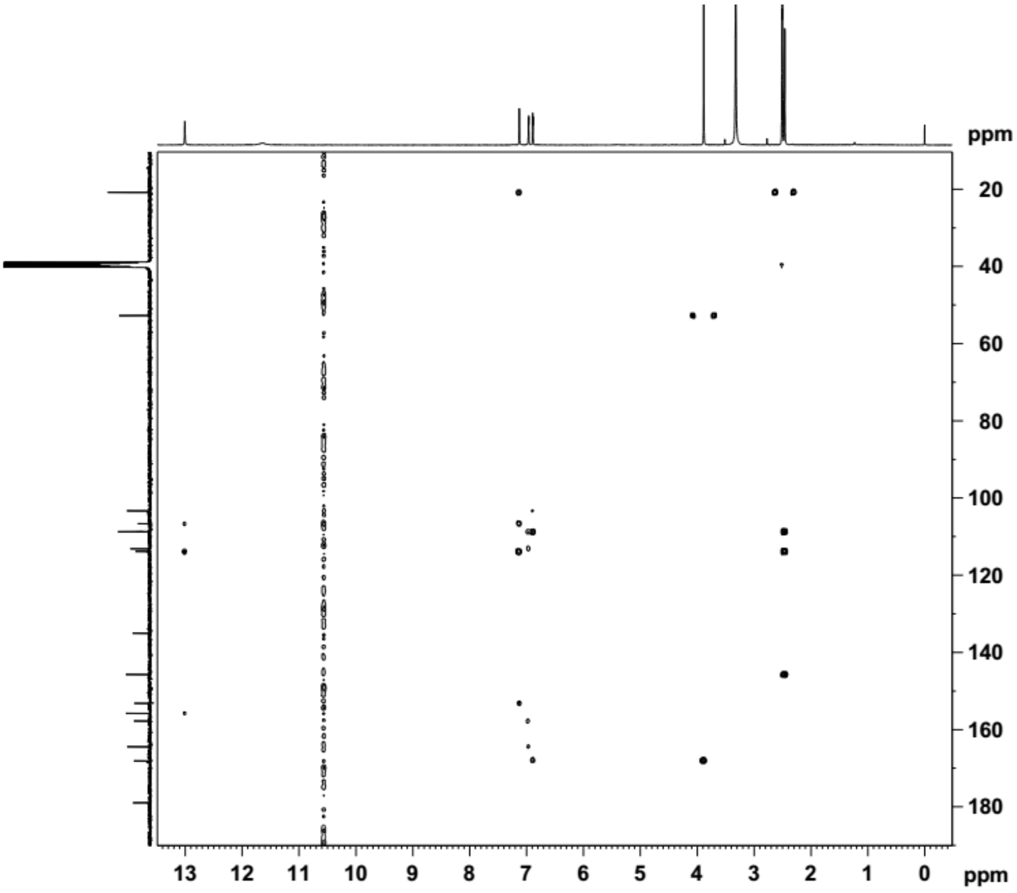

Figure S4. HMBC spectrum of compound **1** in DMSO-d_6

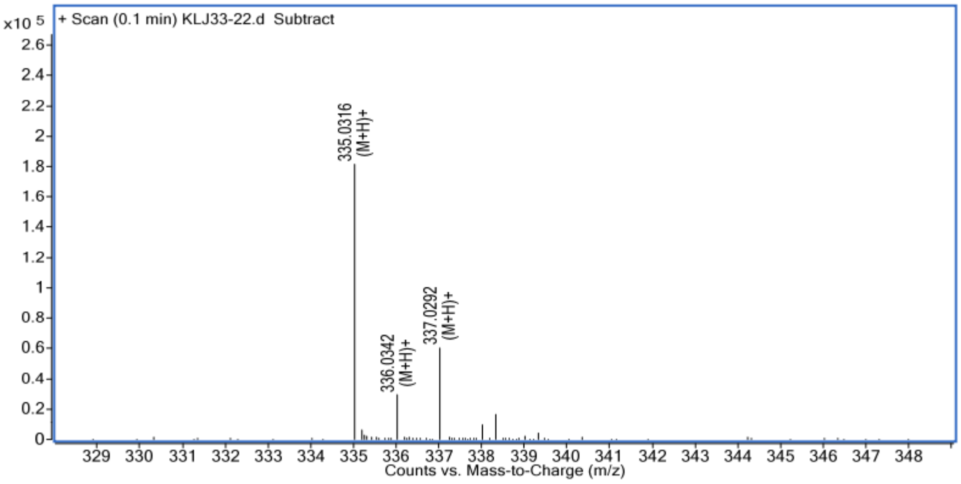

Name	Formula	Score	Mass	Mass (MFG)	Diff (ppm)	Diff (abs. ppm)	Diff (mDa)
	C16 H11 Cl O6	98.84	334.02429	334.02442	0.39	0.39	0.13

Species	Ion Formula	m/z	Height	Score (MFG)	Score (MFG, MS)	Score (MFG, mass)	Score (MFG, abund)	Score (MFG, iso. spacing)
(M+H)+	C16 H12 Cl O6	335.0317	181116	98.84	98.84	99.89	98.65	96.99

m/z	m/z (Calc)	Diff (ppm)	Diff (mDa)	Height	Height (Calc)	Height %	Height % (Calc)	Height Sum %	Height Sum% (Calc)
335.0316	335.0317	0.36	0.1	181116	177545.8	100	100	64	62.8
336.0342	336.0351	2.6	0.9	29449.3	31375.4	16.3	17.7	10.4	11.1
337.0292	337.0294	0.76	0.3	60265	61602.3	33.3	34.7	21.3	21.8
338.0314	338.0325	3.41	1.2	9820.9	10560	5.4	5.9	3.5	3.7
339.0334	339.0346	3.44	1.2	1741.7	1582.4	1	0.9	0.6	0.6
340.0417	340.0372	-13.23	-4.5	240.7	170.5	0.1	0.1	0.1	0.1
341.0395	341.0396	0.25	0.1	218.3	15.7	0.1	0	0.1	0

Figure S5. HRESIMS spectrum of compound **1**

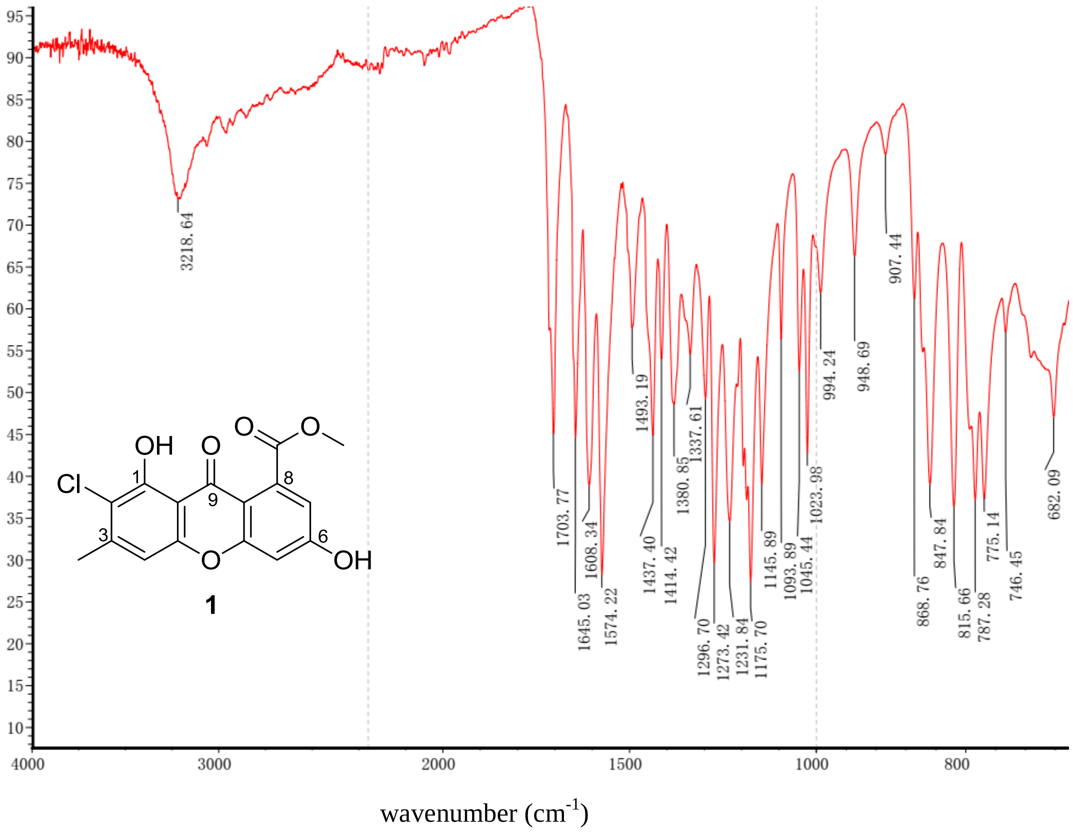

Figure S6. IR spectrum of compound **1**

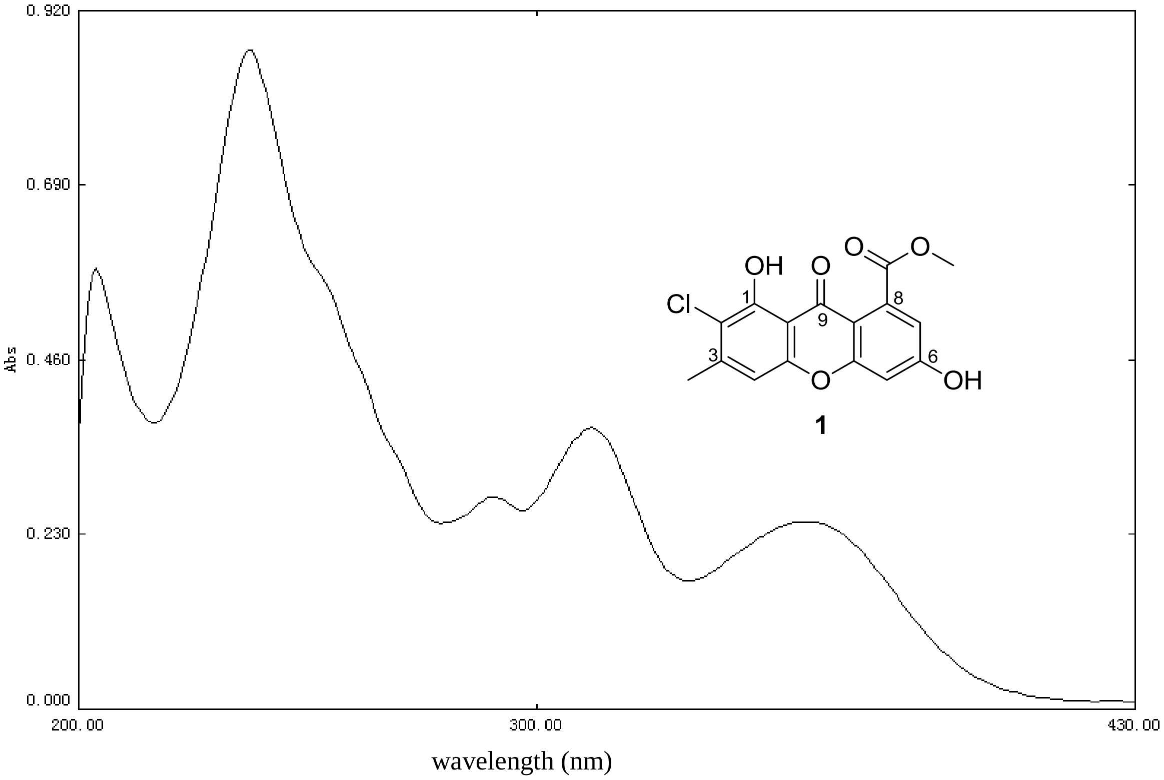

Figure S7. UV spectrum of compound **1** in MeOH

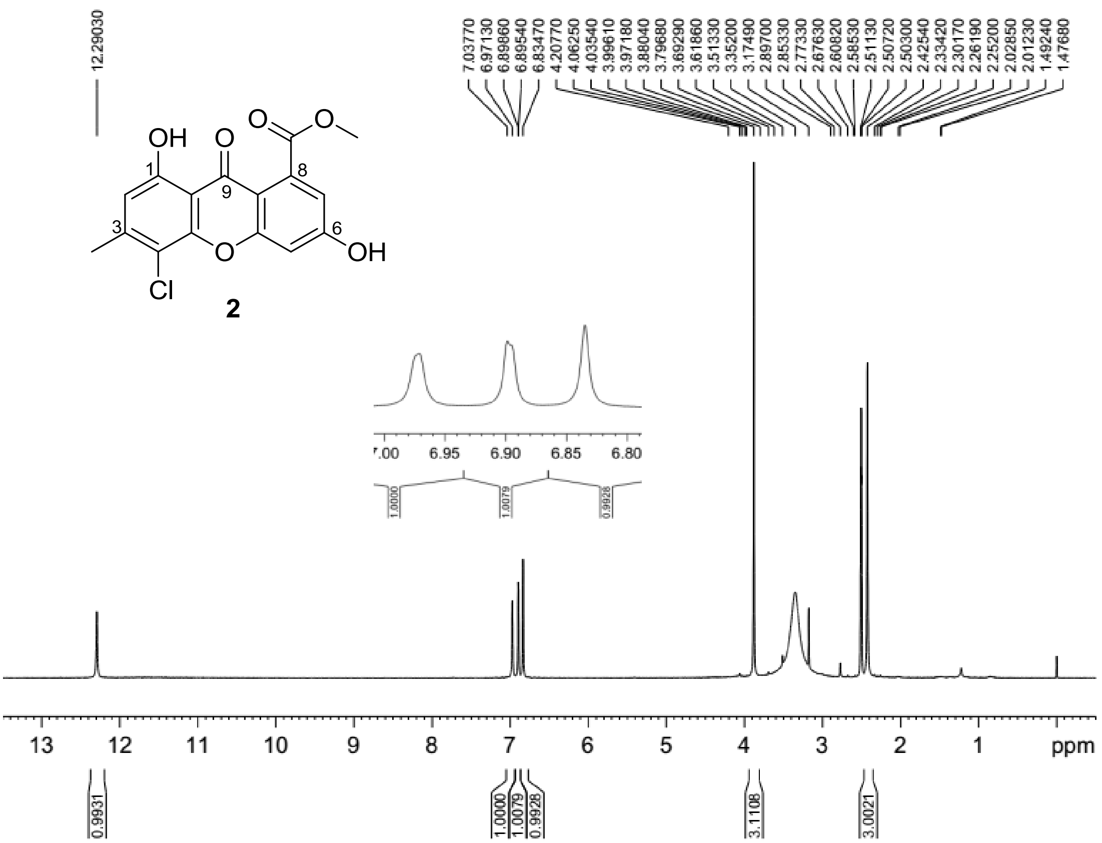

Figure S8. ^{1}H NMR spectrum (400 MHz) of compound **2** in DMSO-d_6

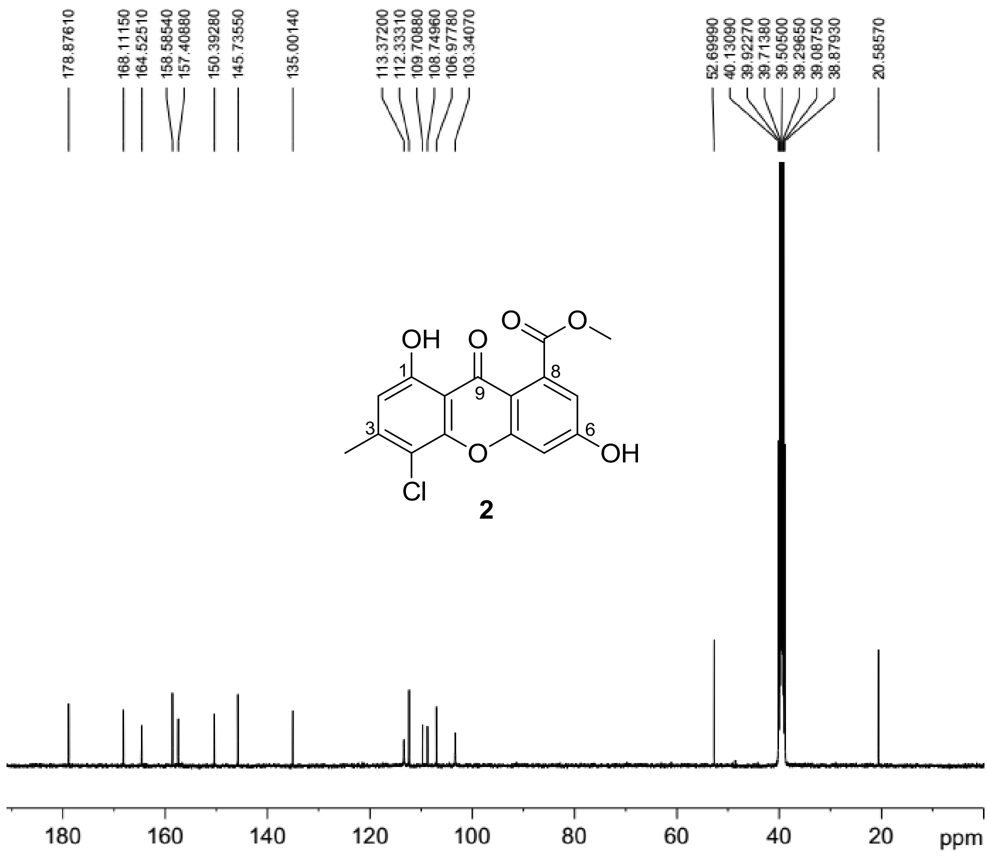

Figure S9. ^{13}C NMR spectrum (100 MHz) of compound **2** in DMSO-d_6

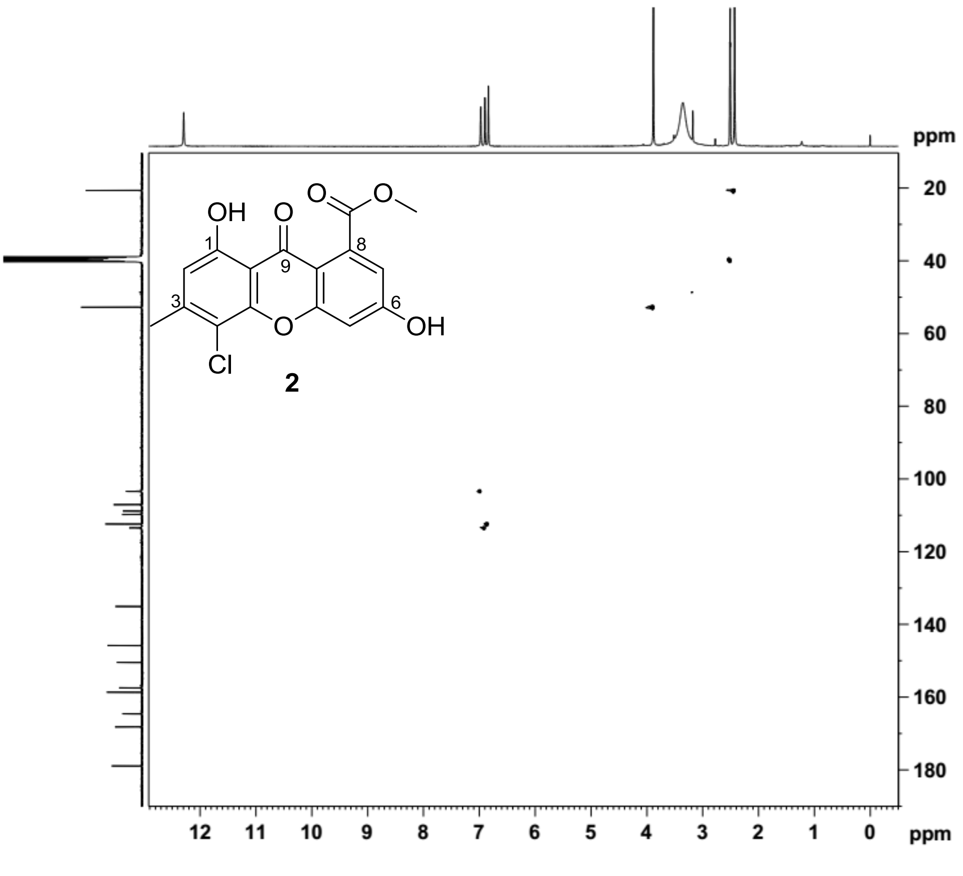

Figure S10. HSQC spectrum of compound **2** in DMSO-d_6

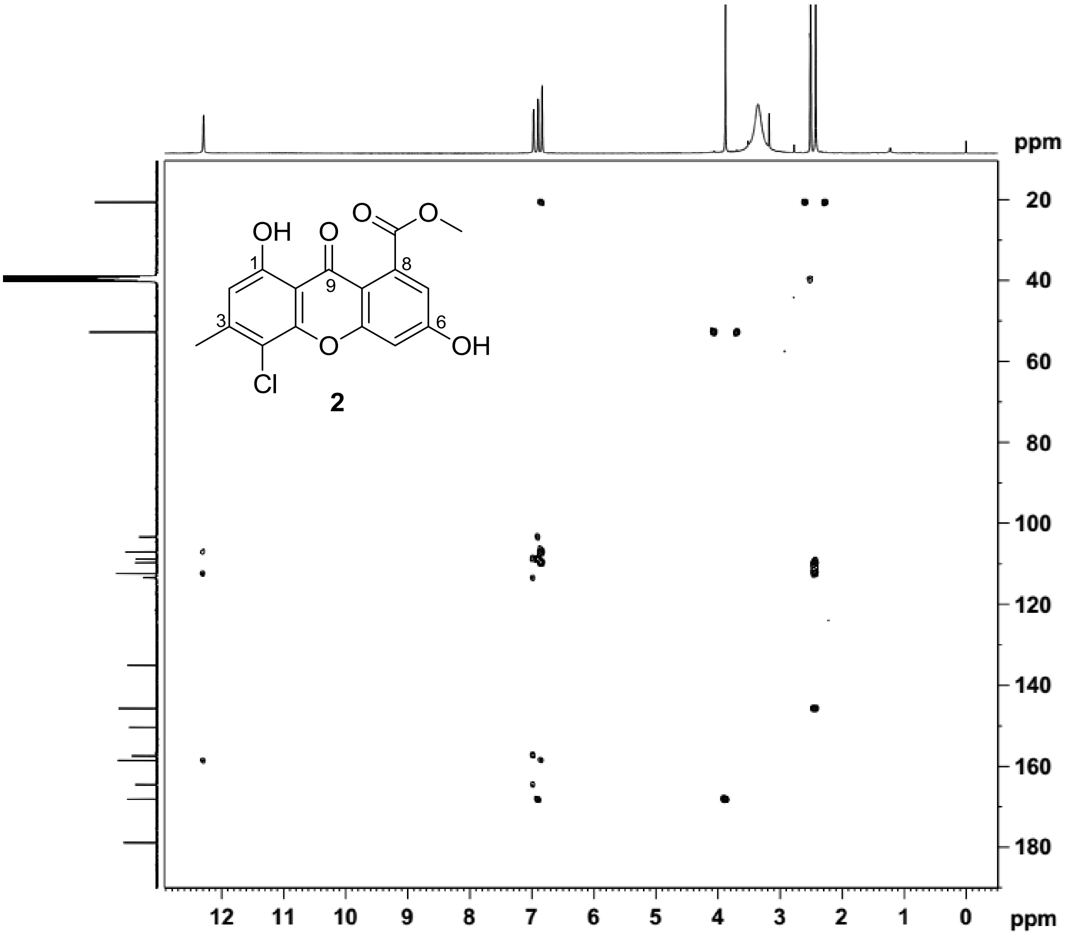

Figure S11. HMBC spectrum of compound **2** in DMSO-d_6

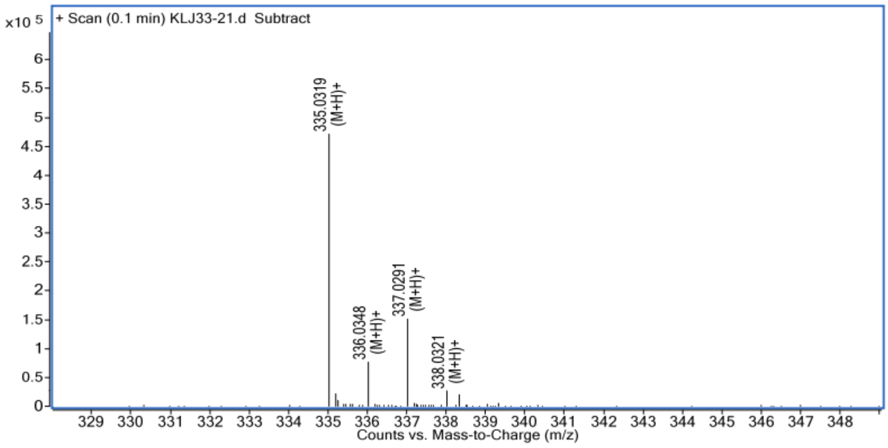

Name	Formula	Score	Mass	Mass (MFG)	Diff (ppm)	Diff (abs. ppm)	Diff (mDa)
	C16 H11 Cl O6	98.68	334.02459	334.02442	-0.51	0.51	-0.17

Species	Ion Formula	m/z	Height	Score (MFG)	Score (MFG, MS)	Score (MFG, mass)	Score (MFG, abund)	Score (MFG, iso. spacing)
(M+H)+	C16 H12 Cl O6	335.0317	471504	98.68	98.68	99.81	97.14	98.26

m/z	m/z (Calc)	Diff (ppm)	Diff (mDa)	Height	Height (Calc)	Height %	Height % (Calc)	Height Sum %	Height Sum% (Calc)
335.0319	335.0317	-0.56	-0.2	471504	457787.1	100	100	64.7	62.8
336.0348	336.0351	0.74	0.2	76686.8	80898.9	16.3	17.7	10.5	11.1
337.0291	337.0294	1.06	0.4	150598.5	158836.5	31.9	34.7	20.7	21.8
338.0321	338.0325	1.24	0.4	26115.1	27228	5.5	5.9	3.6	3.7
339.036	339.0346	-4.09	-1.4	3890.8	4080	0.8	0.9	0.5	0.6
340.0331	340.0372	11.96	4.1	474.8	439.6	0.1	0.1	0.1	0.1

Figure S12. HRESIMS spectrum of compound **2**

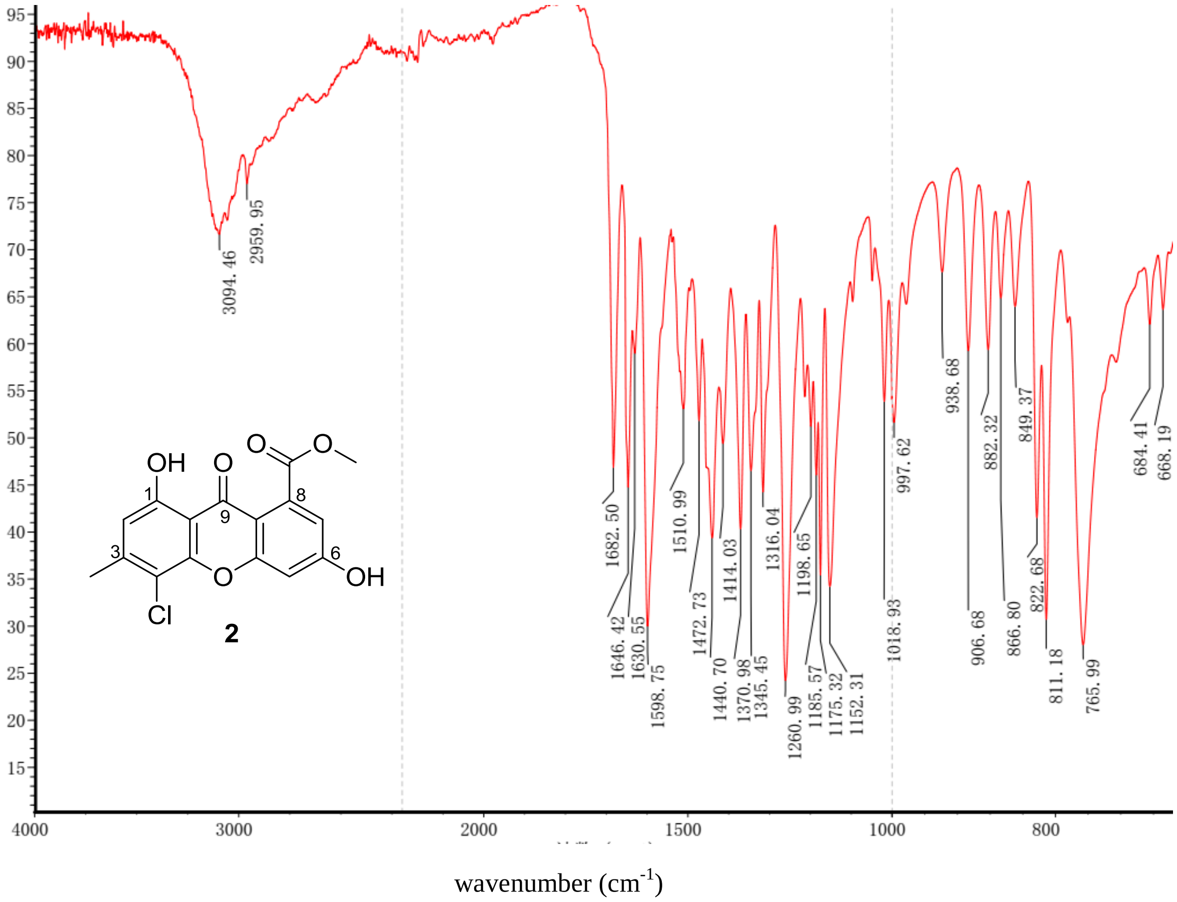

Figure S13. IR spectrum of compound **2**

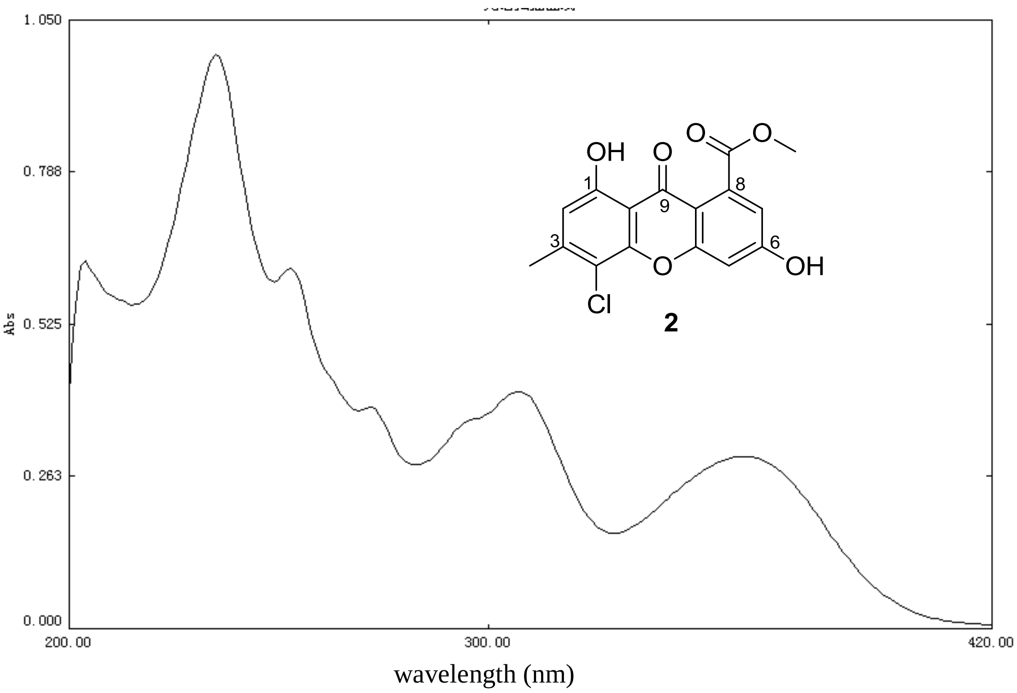

Figure S14. UV spectrum of compound **2** in MeOH

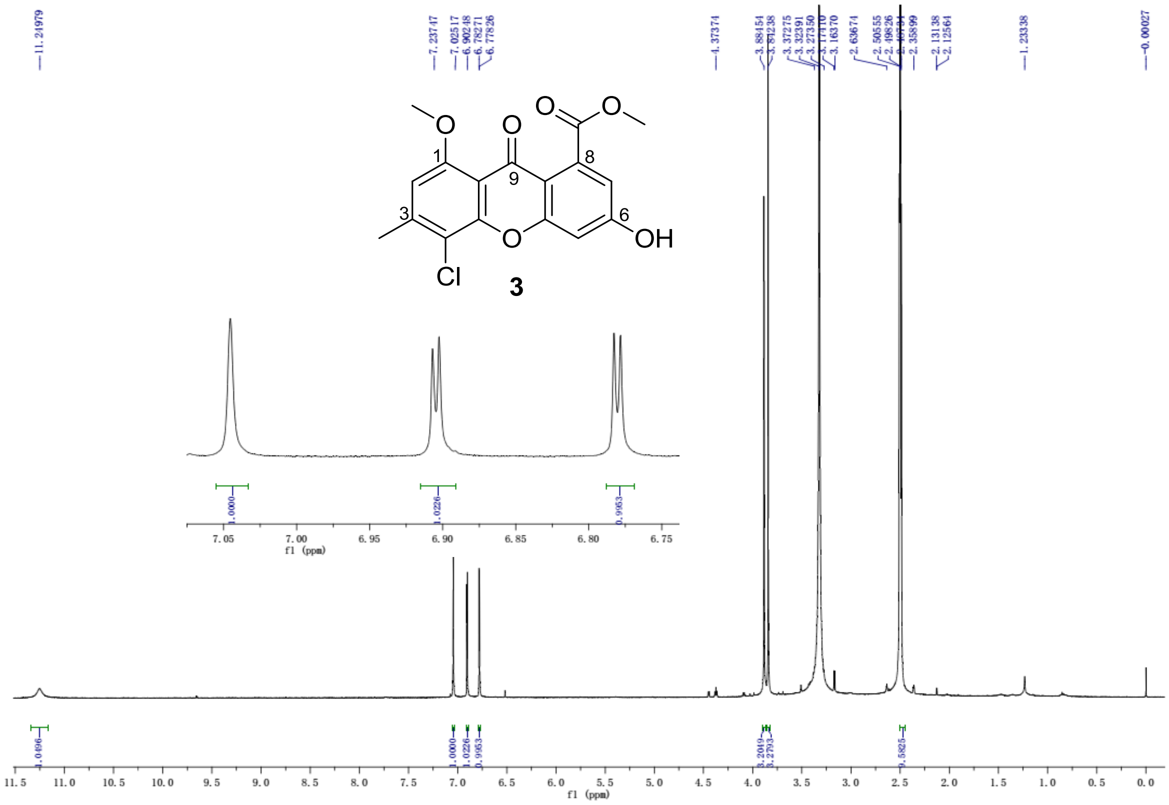

Figure S15. ^{1}H NMR spectrum (500 MHz) of compound **3** in DMSO-d_6

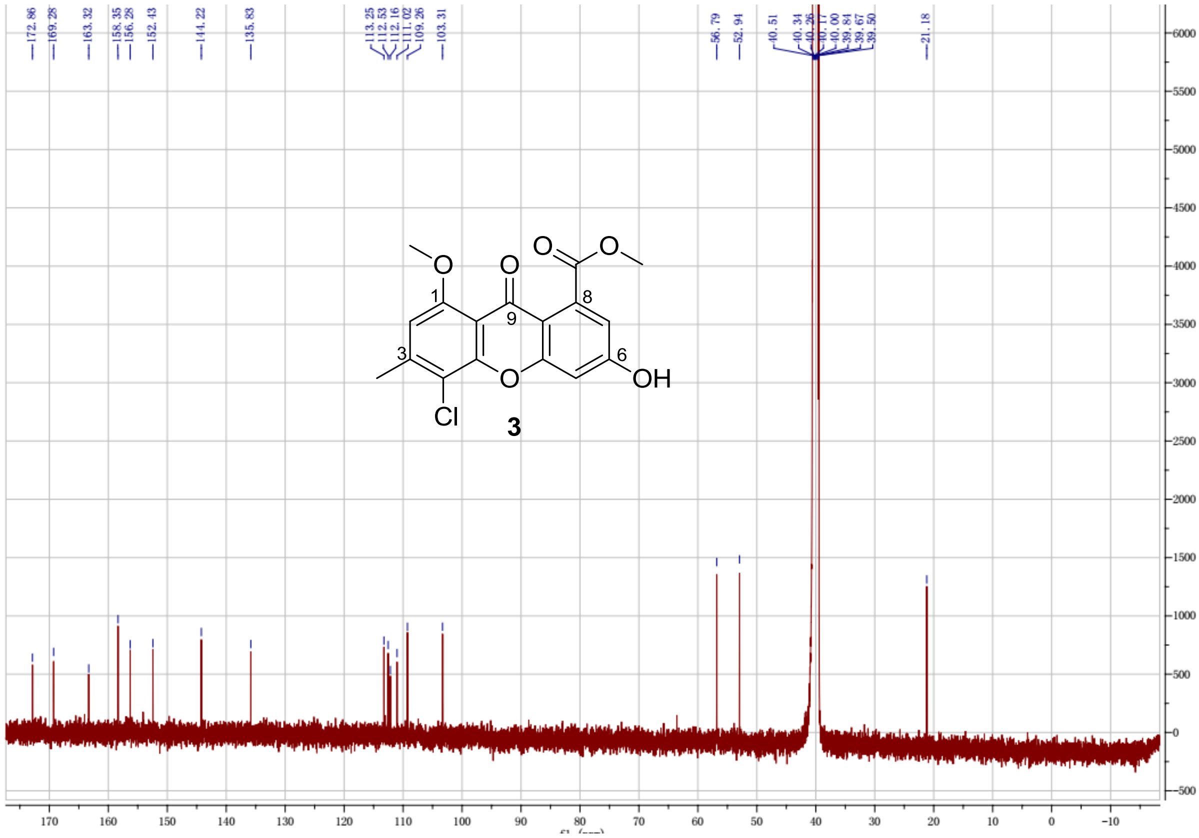

Figure S16. ^{13}C NMR spectrum (125 MHz) of compound **3** in DMSO-d_6

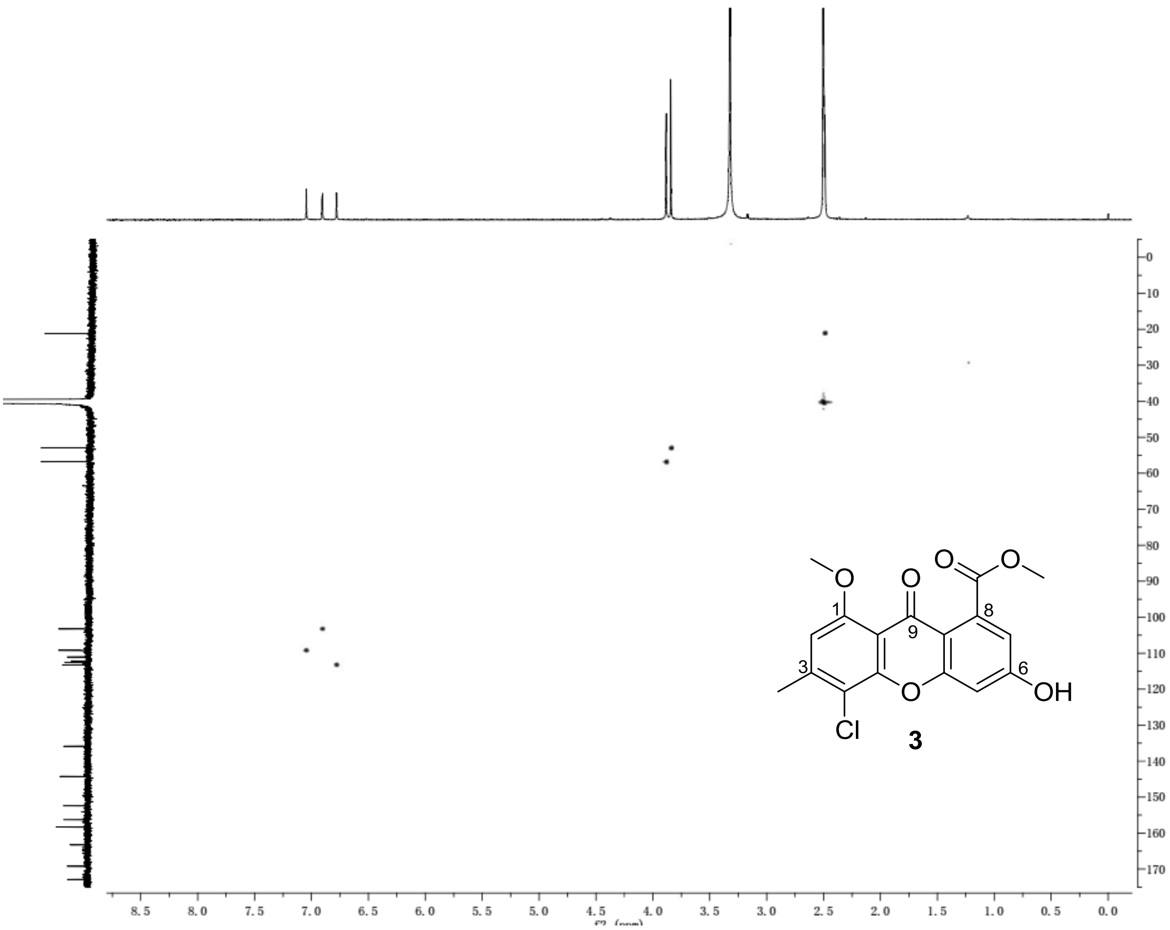

Figure S17. HSQC spectrum of compound **3** in DMSO-d_6

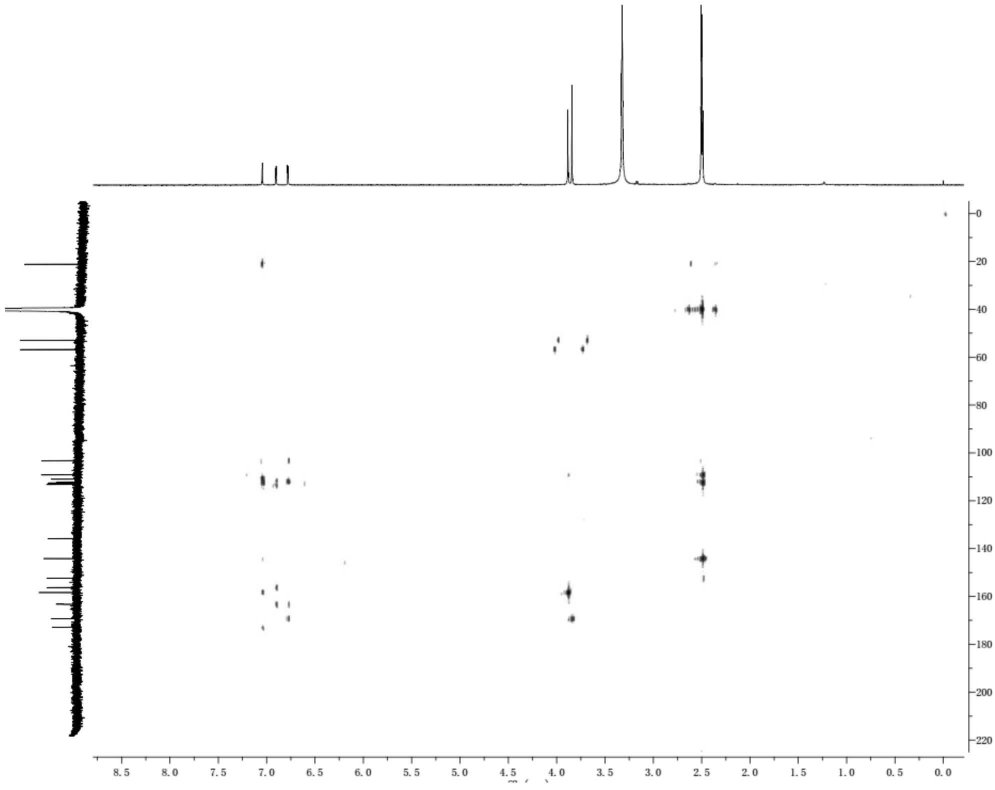

Figure S18. HMBC spectrum of compound **3** in DMSO-d_6

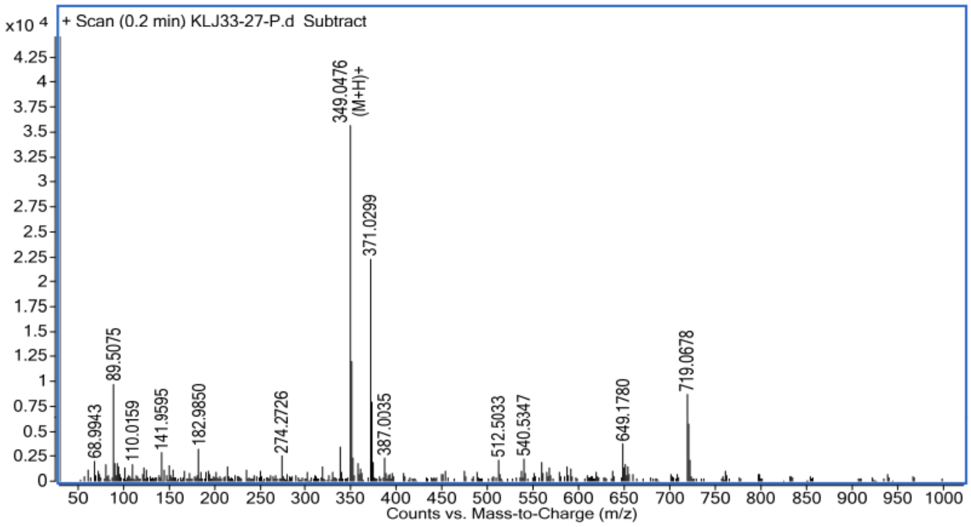

Formula	Score	Mass	Mass (MFG)	Diff (ppm)	Diff (abs. ppm)	Diff (mDa)	ID Source	Score (MFG)	DBE
C17 H13 Cl O6	98.75	348.04035	348.04007	-0.82	0.82	-0.29	MFG	98.75	11

Species	Ion Formula	m/z	Height	Score (MFG)	Score (MFG, MS	Score (MFG, mass	Score (MFG, abund	core (MFG, iso. spacing)
(M+H)+	C17 H14 Cl O6	349.0473	35665.6	98.75	98.75	99.48	97.73	98.5

m/z	m/z (Calc)	Diff (ppm)	Diff (mDa)	Height	Height (Calc)	Height %	Height % (Calc)	Height Sum %	Height Sum% (Calc)
349.0476	349.0473	-0.74	-0.3	35665.6	35584.1	100	100	62.3	62.1
350.0512	350.0507	-1.38	-0.5	7129.5	6681.4	20	18.8	12.4	11.7
351.0461	351.0451	-2.83	-1	11947.6	12416	33.5	34.9	20.9	21.7
352.0461	352.0482	5.85	2.1	2260.3	2252.8	6.3	6.3	3.9	3.9
353.0494	353.0504	2.77	1	271.9	340.6	0.8	1	0.5	0.6

Figure S19. HRESIMS spectrum of compound **3**

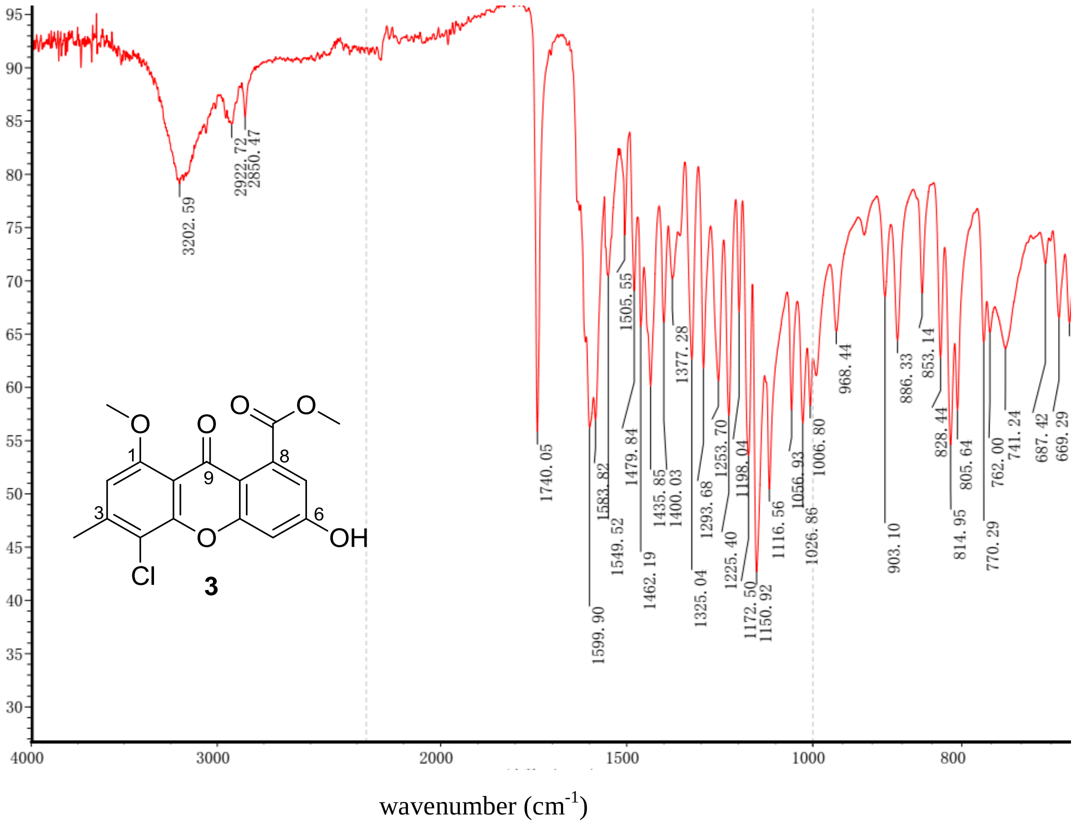

Figure S20. IR spectrum of compound **3**

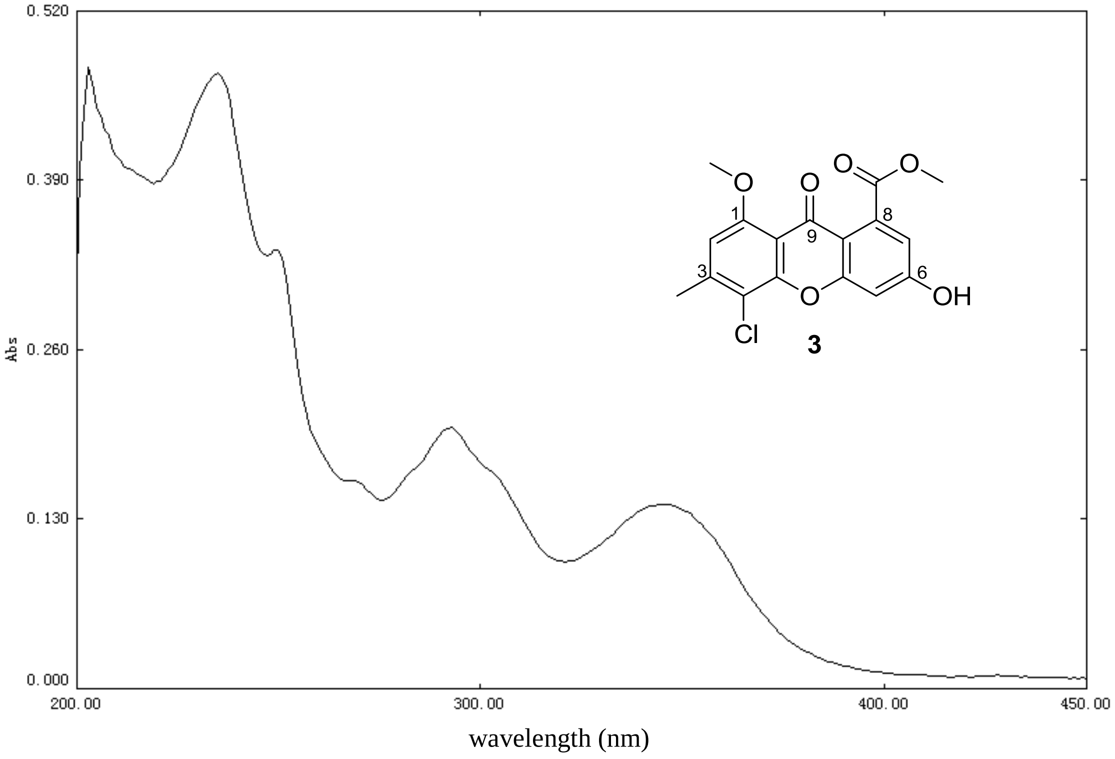

Figure S21. UV spectrum of compound **3** in MeOH

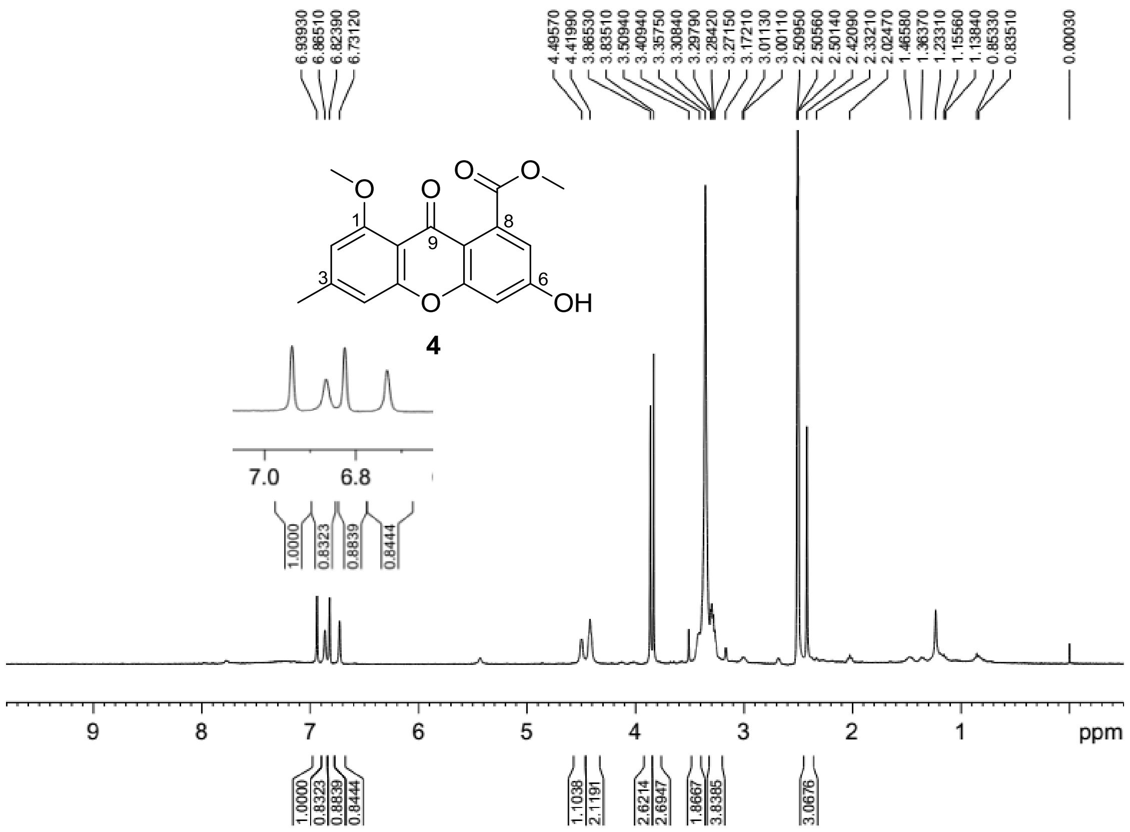

Figure S22. ^{1}H NMR spectrum (400 MHz) of compound **4** in DMSO-d_6

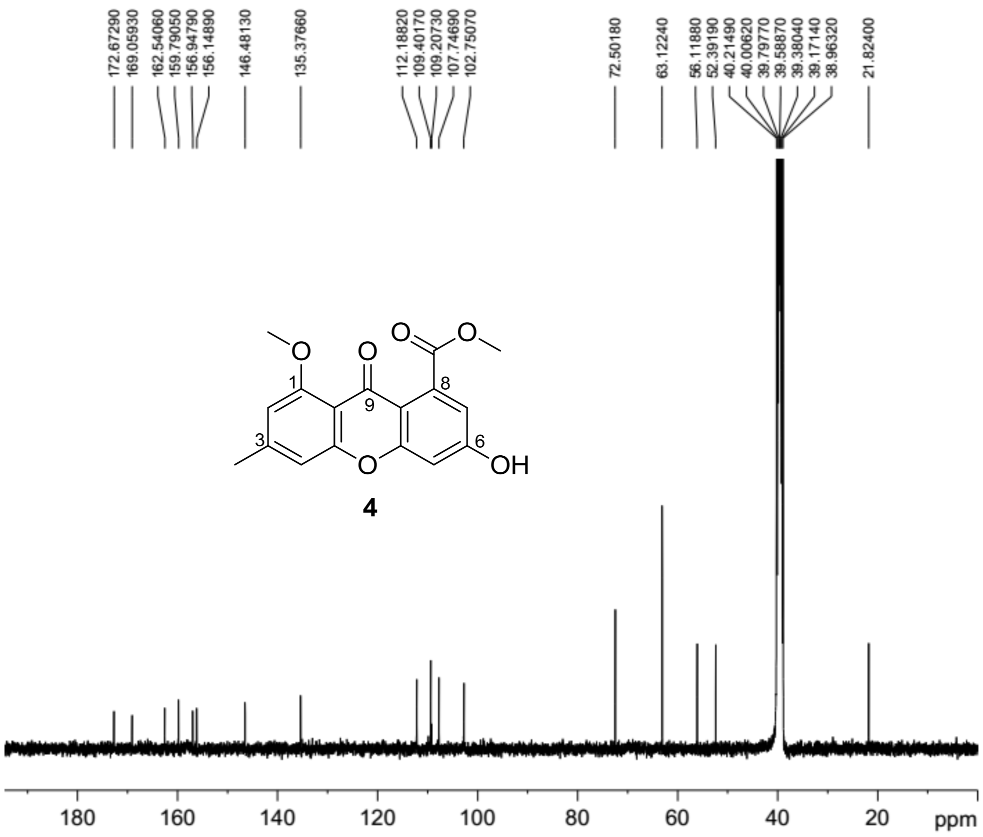

Figure S23. ^{13}C NMR spectrum (100 MHz) of compound **4** in DMSO-d_6

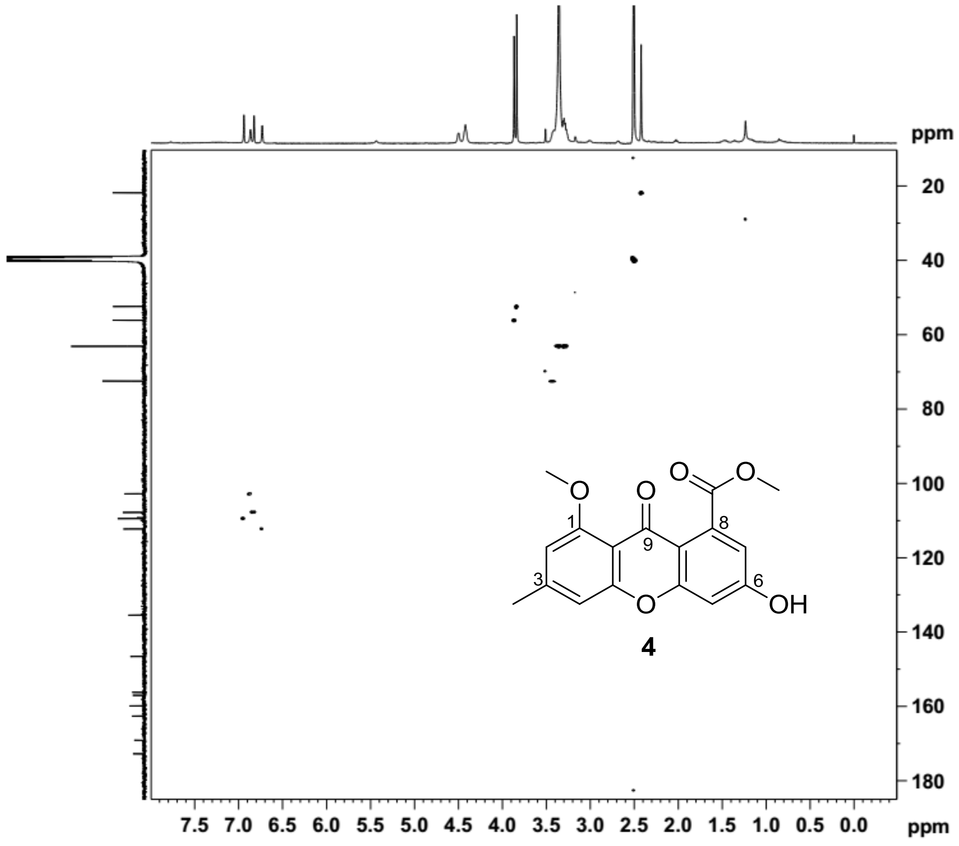

Figure S24. HSQC spectrum of compound **4** in DMSO-d_6

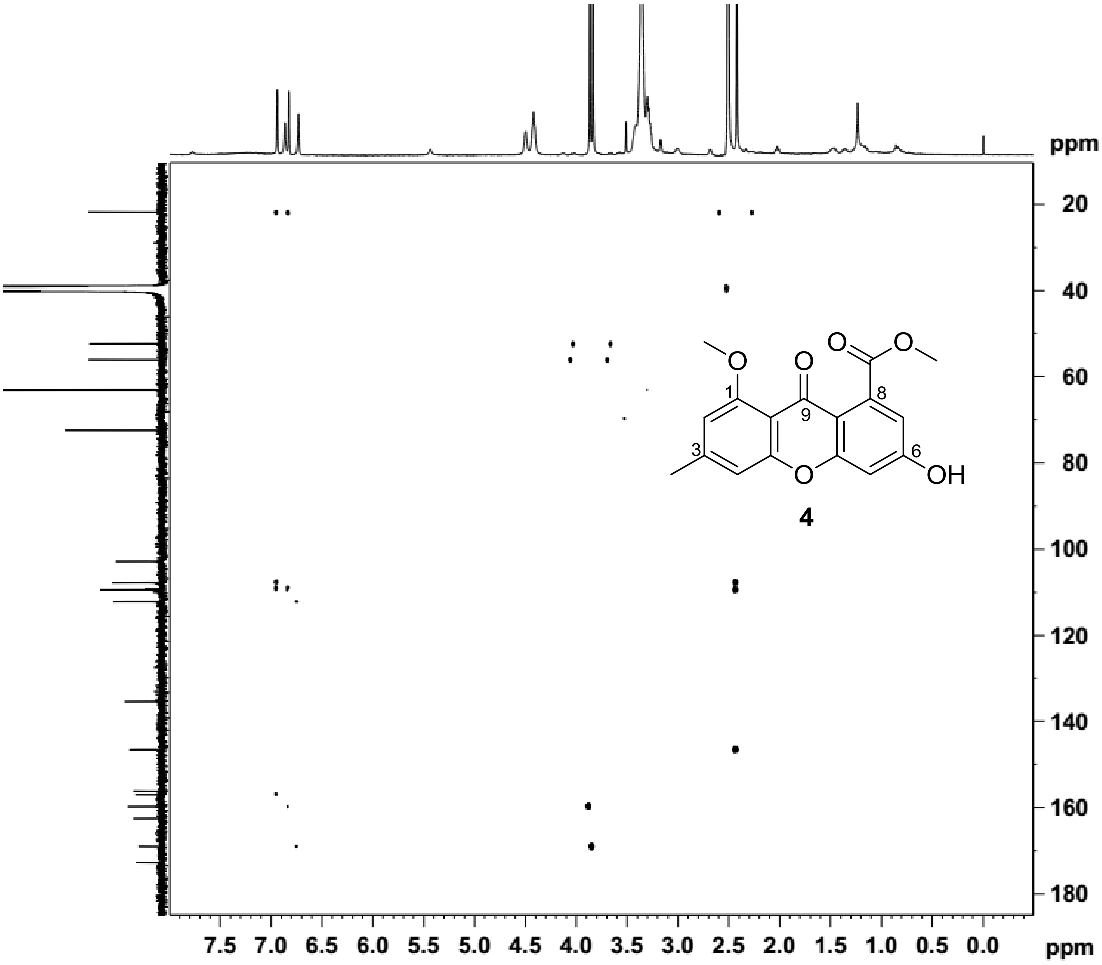

Figure S25. HMBC spectrum of compound **4** in DMSO-d_6

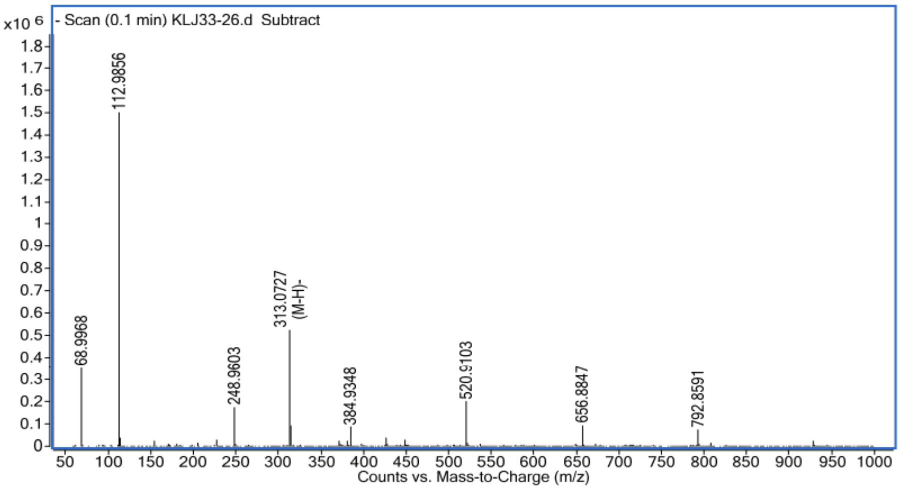

Formula	Score	Mass	Mass (MFG)	Diff (ppm)	Diff (abs. ppm)	Diff (mDa)	ID Source	Score (MFG)	DBE
C17 H14 O6	95.75	314.07996	314.07904	-2.93	2.93	-0.92	MFG	95.75	11

Species	Ion Formula	m/z	Height	Score (MFG)	Score (MFG, MS)	Score (MFG, mass)	Score (MFG, abund)	Score (MFG, iso. spacing)
(M-H)-	C17 H13 O6	313.0718	519700.7	95.75	95.75	94.17	99.21	94.75

m/z	m/z (Calc)	Diff (ppm)	Diff (mDa)	Height	Height (Calc)	Height %	Height % (Calc)	Height Sum %	Height Sum% (Calc)
313.0727	313.0718	-2.96	-0.9	519700.7	514412.8	100	100	83	82.2
314.0751	314.0752	0.07	0	92719.3	96528.6	17.8	18.8	14.8	15.4
315.0769	315.0775	1.81	0.6	13409	14887.6	2.6	2.9	2.1	2.4

Figure S26. HRESIMS spectrum of compound **4**

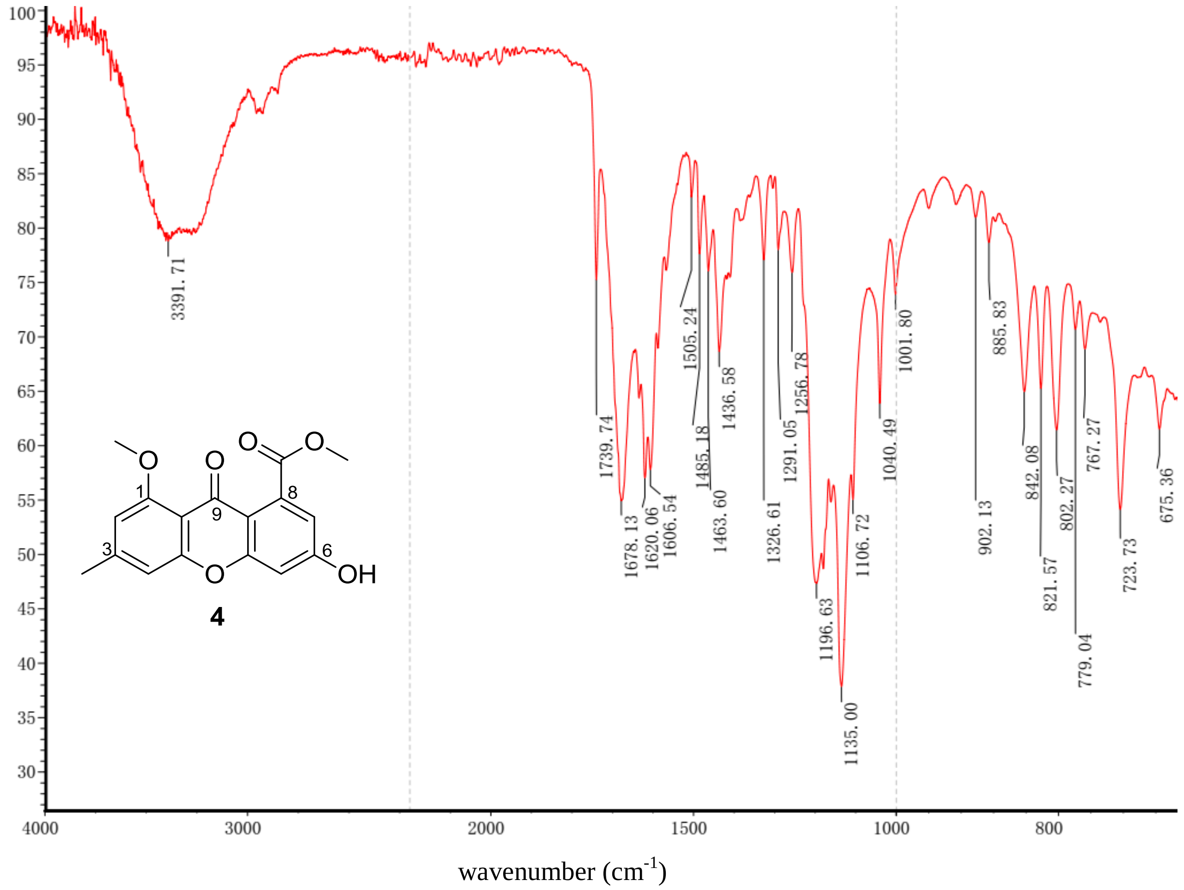

Figure S27. IR spectrum of compound **4**

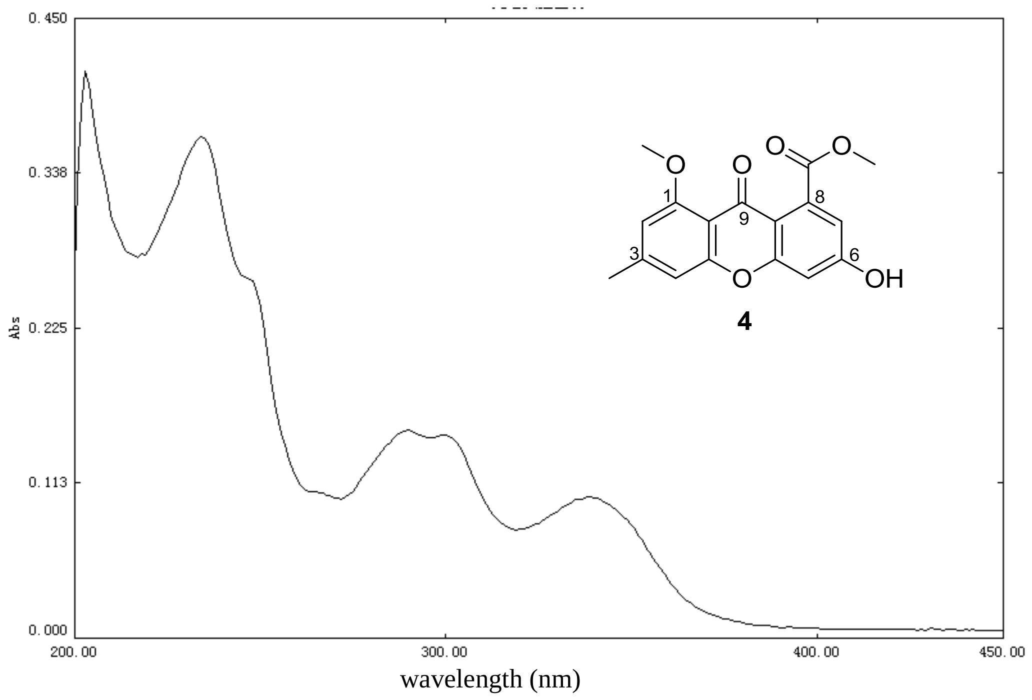

Figure S28. UV spectrum of compound **4** in MeOH

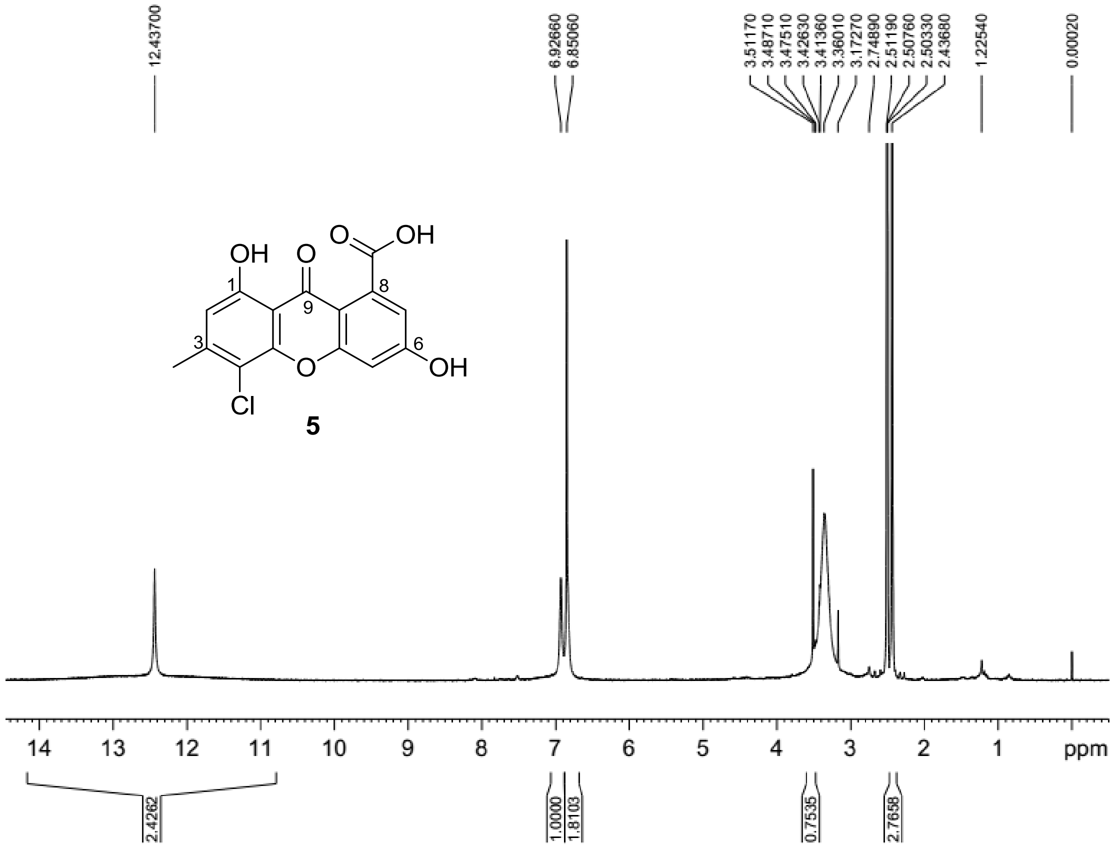

Figure S29. ^{1}H NMR spectrum (400 MHz) of compound **5** in DMSO-d_6

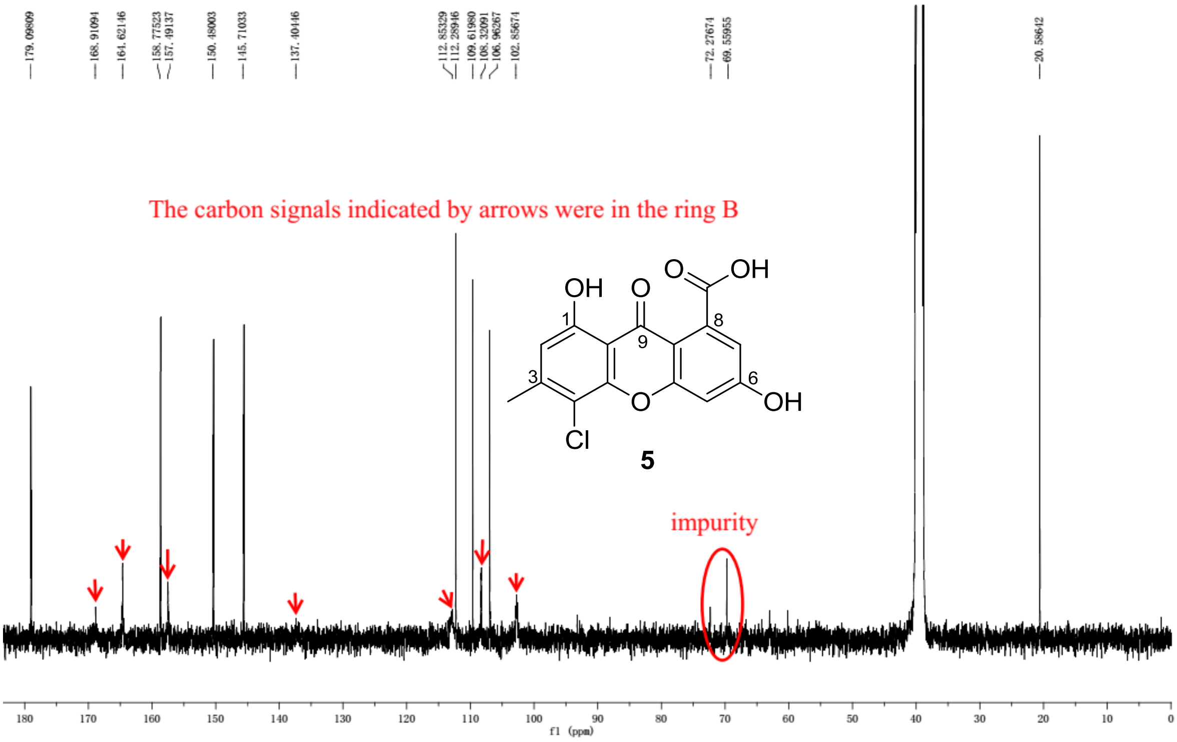

Figure S30. ^{13}C NMR spectrum (100 MHz) of compound **5** in DMSO-d_6

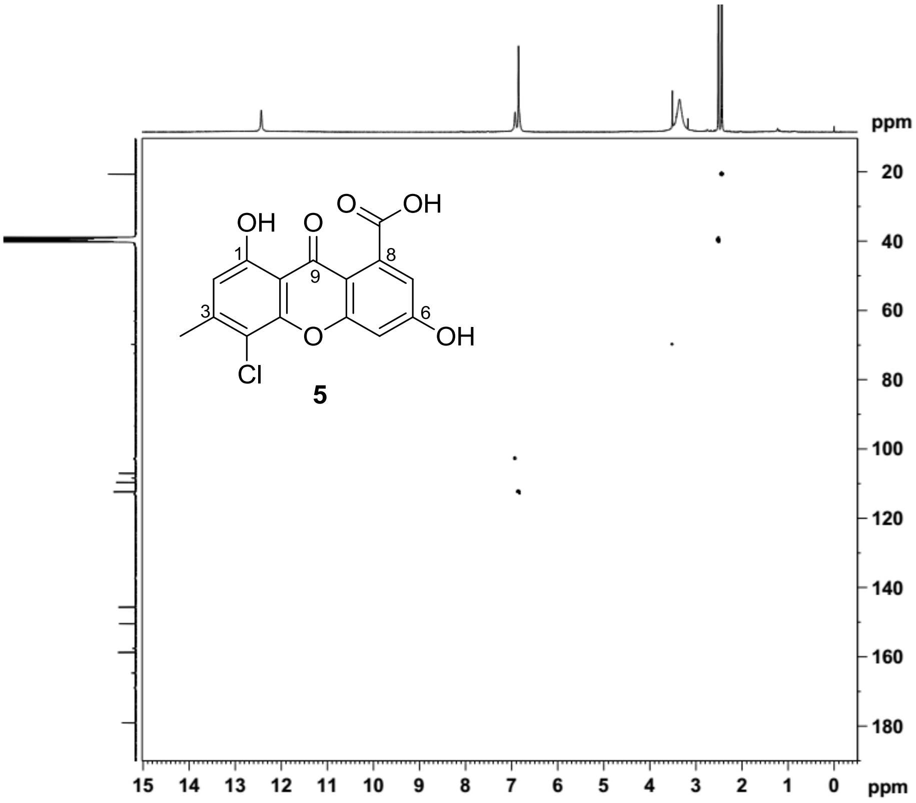

Figure S31. HSQC spectrum of compound **5** in DMSO-d_6

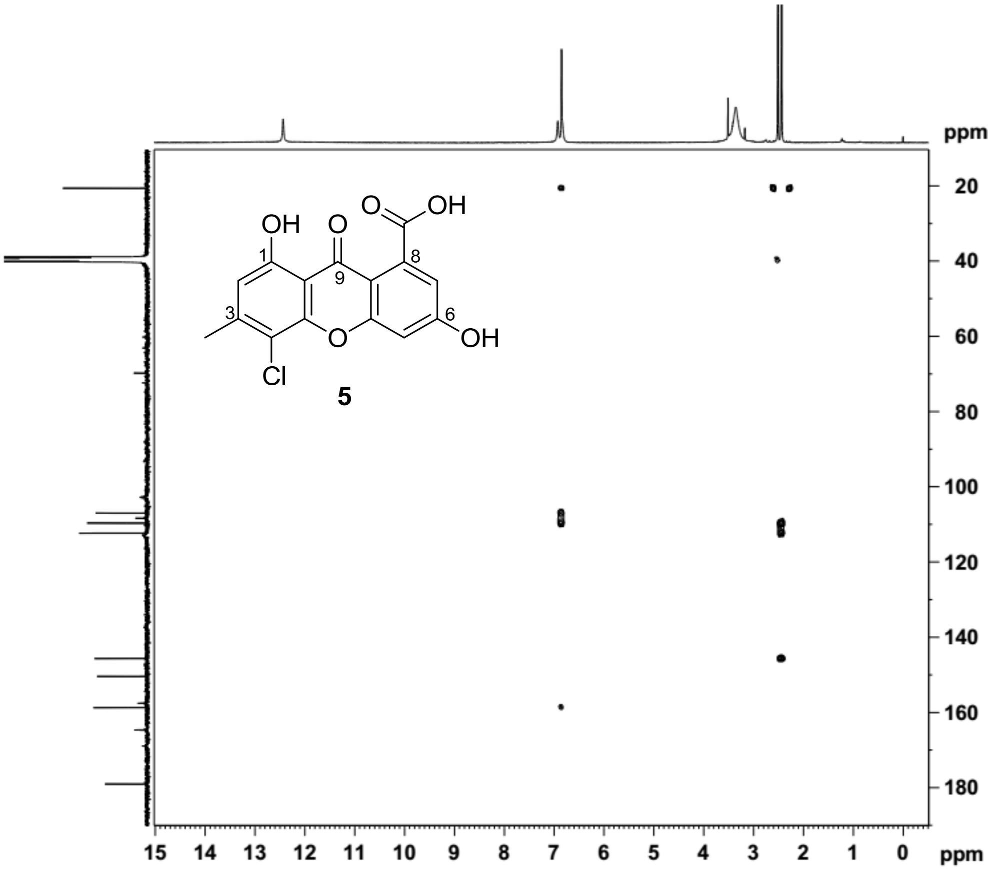

Figure S32. HMBC spectrum of compound **5** in DMSO-d_6

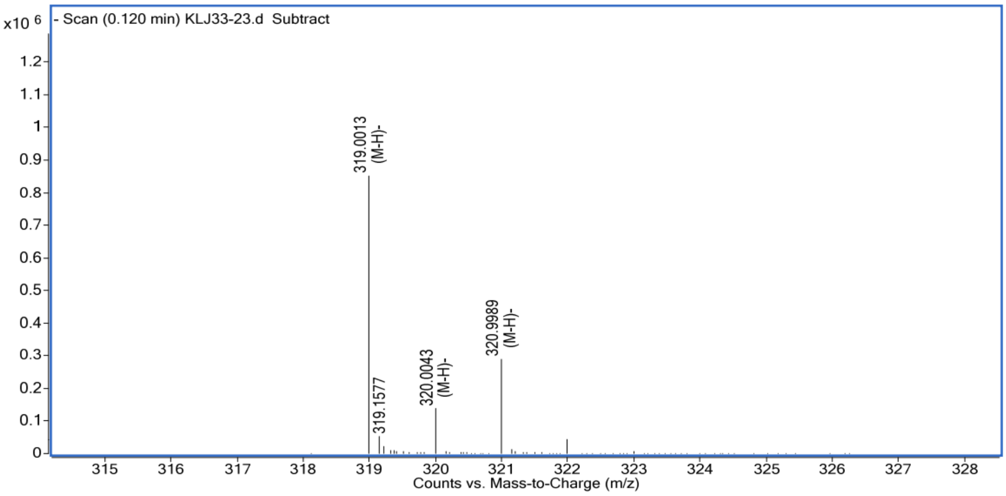

Name	Formula	Score	Mass	Mass (MFG)	Diff (ppm)	Diff (abs. ppm)	Diff (mDa)
	C15 H9 Cl O6	99.61	320.0086	320.0088	0.47	0.47	0.15

Species	Ion Formula	m/z	Height	Score (MFG)	Score (MFG, MS)	Score (MFG, mass)	Score (MFG, abund)	Score (MFG, iso. spacing)
(M-H)-	C15 H8 Cl O6	319.0015	852349.3	99.61	99.61	99.84	99.78	98.93

m/z	m/z (Calc)	Diff (ppm)	Diff (mDa)	Height	Height (Calc)	Height %	Height % (Calc)	Height Sum %	Height Sum% (Calc)
319.0013	319.0015	0.45	0.1	852349.3	844113.5	100	100	64.1	63.5
320.0043	320.0049	1.79	0.6	136953	139651.5	16.1	16.5	10.3	10.5
320.9989	320.9992	0.83	0.3	289426.4	291299.7	34	34.5	21.8	21.9
322.0008	322.0023	4.73	1.5	43583.8	46920.3	5.1	5.6	3.3	3.5
323.0032	323.0043	3.36	1.1	6468	6992.7	0.8	0.8	0.5	0.5
324.005	324.0069	6.01	1.9	928.6	731.6	0.1	0.1	0.1	0.1

Figure S33. HRESIMS spectrum of compound **5**

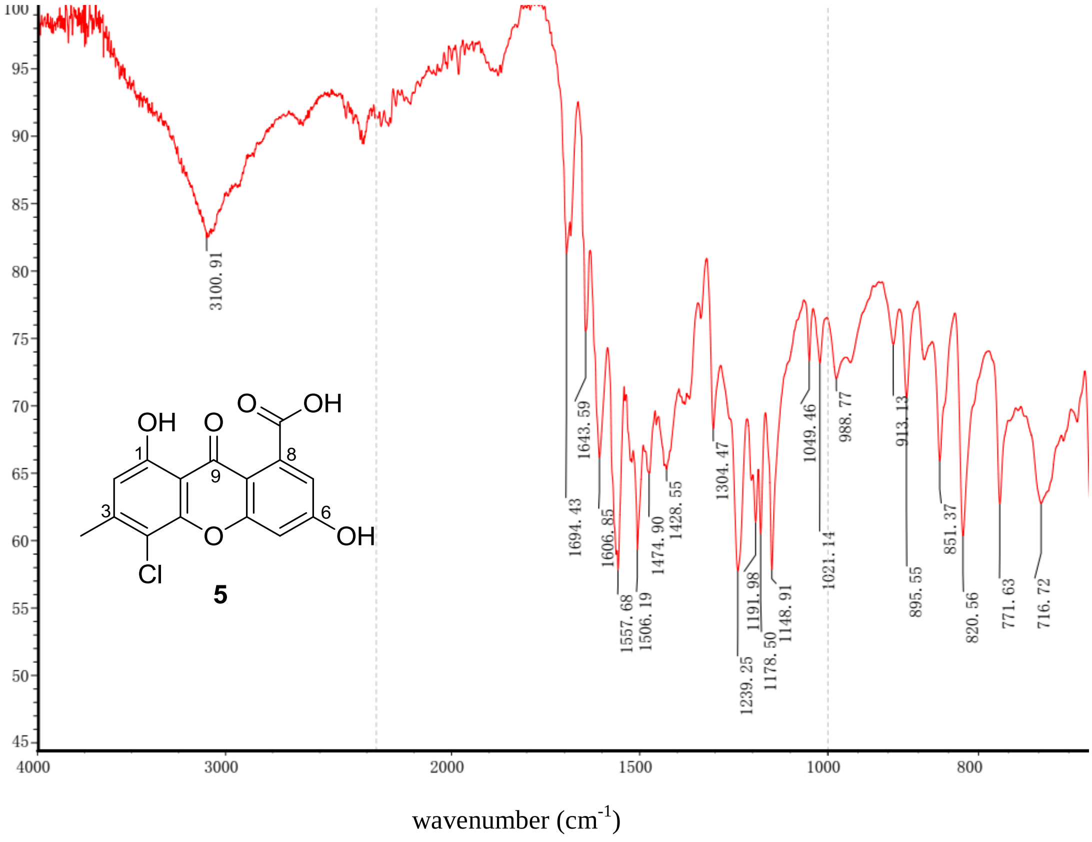

Figure S34. IR spectrum of compound **5**

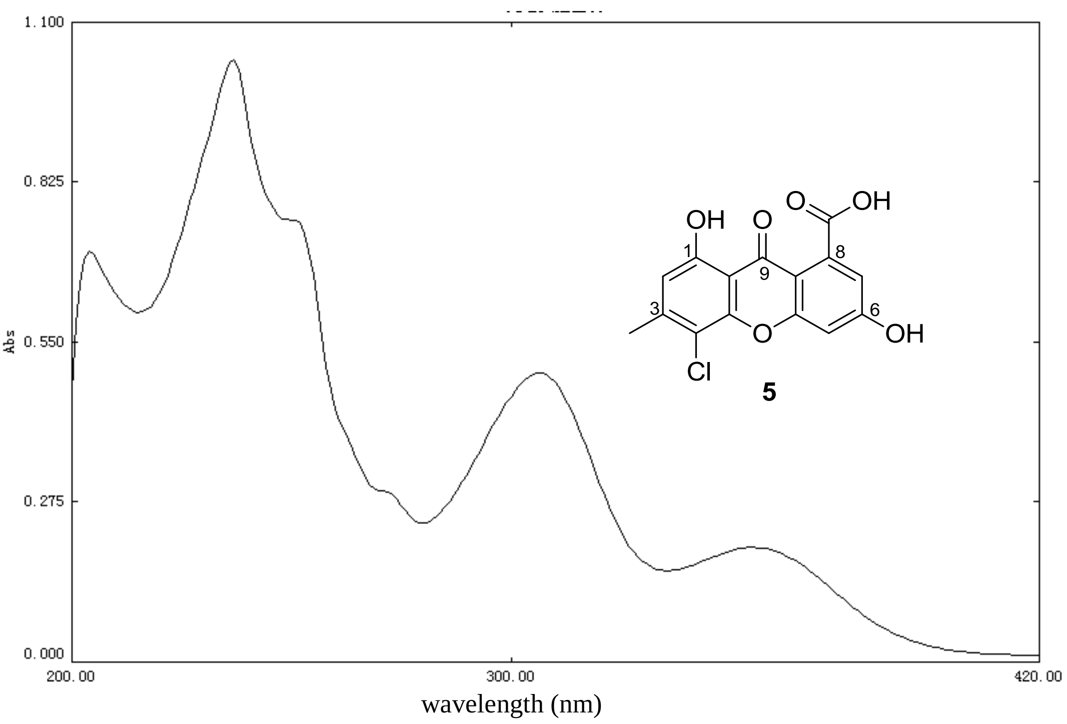

Figure S35. UV spectrum of compound **5** in MeOH

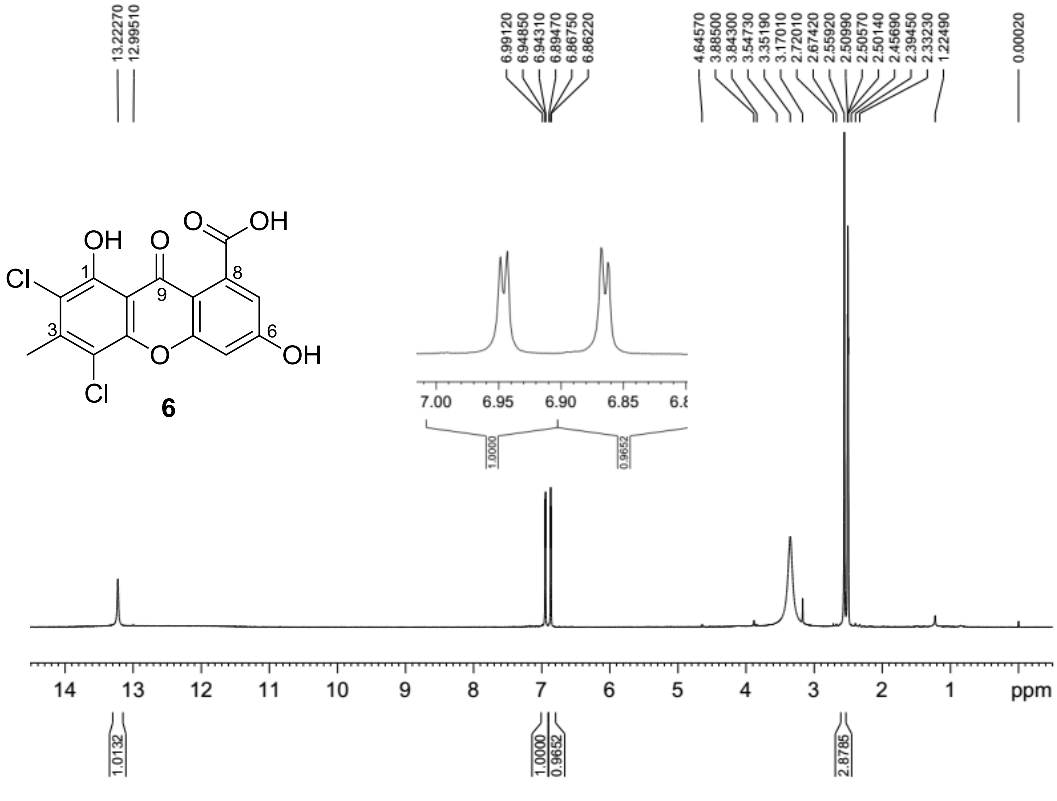

Figure S36. ^{1}H NMR spectrum (400 MHz) of compound **6** in DMSO-d_6

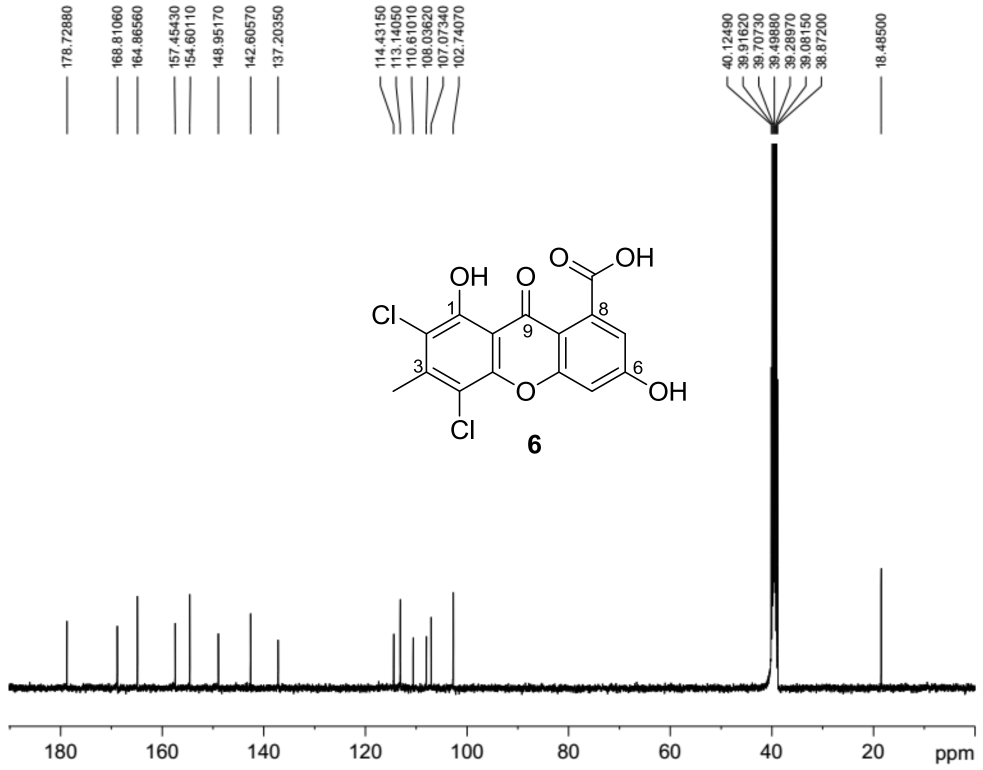

Figure S37. ^{13}C NMR spectrum (100 MHz) of compound **6** in DMSO-d_6

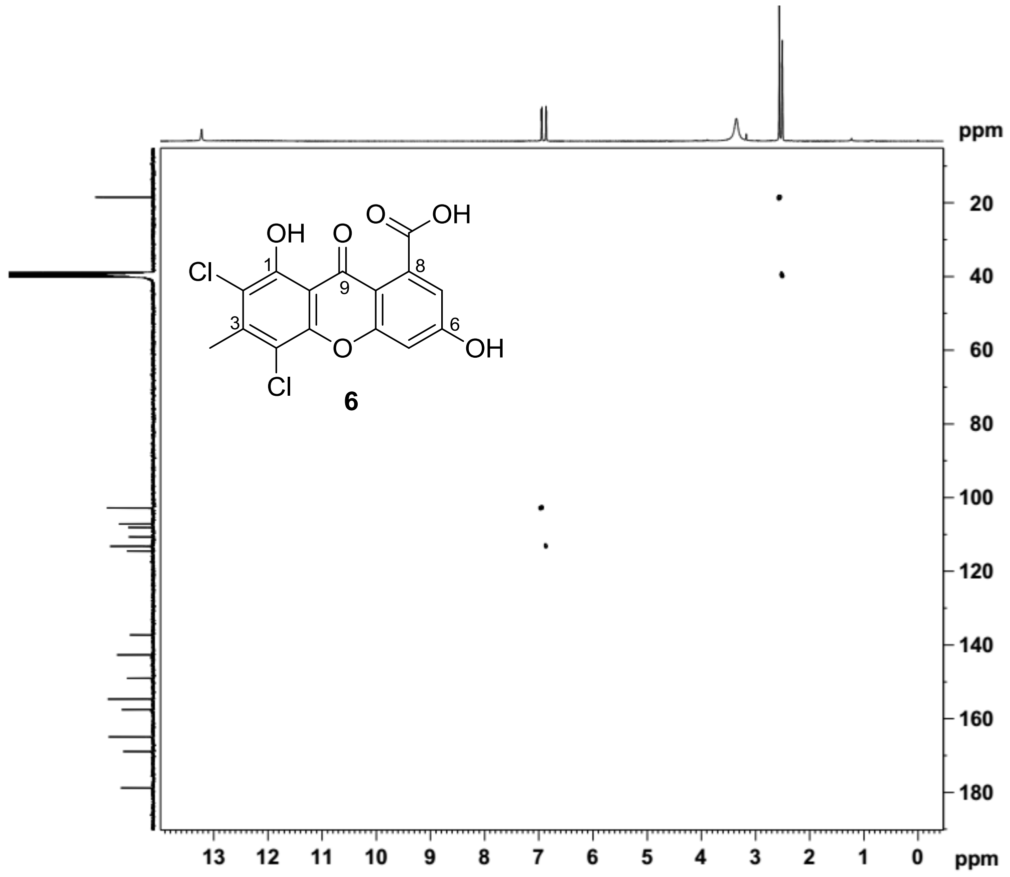

Figure S38. HSQC spectrum of compound **6** in DMSO-d_6

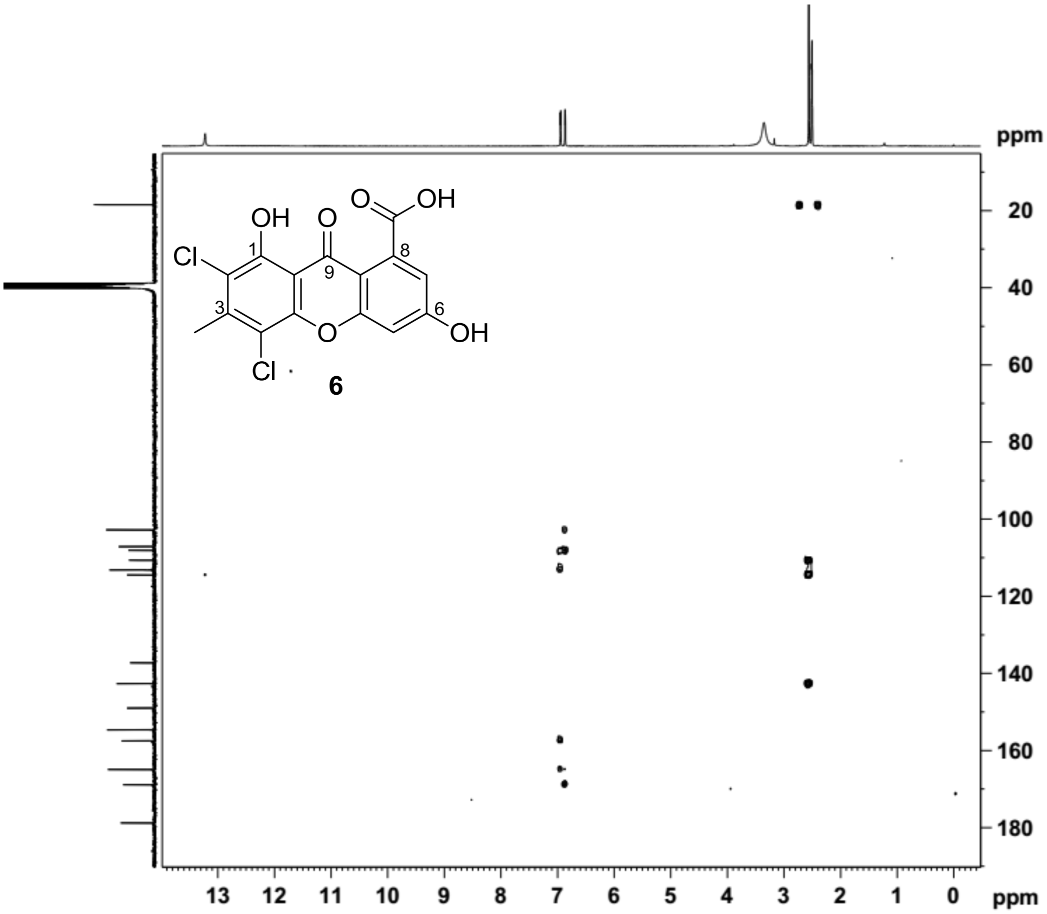

Figure S39. HMBC spectrum of compound **6** in DMSO-d_6

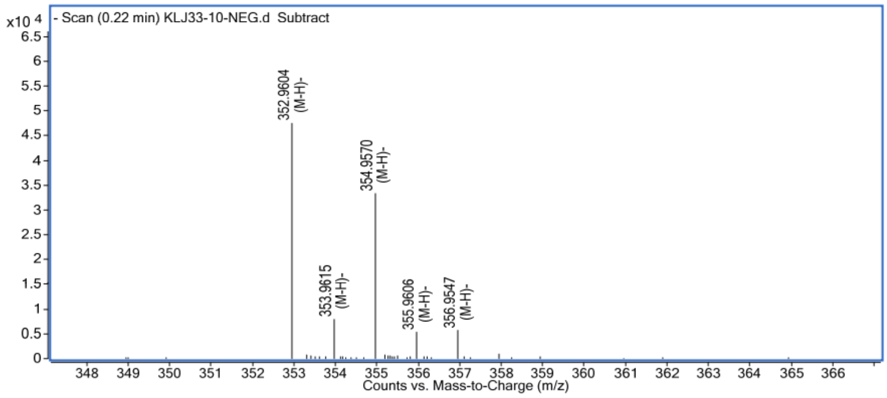

Name	Formula	Score	Mass	Mass (DB)	Mass (MFG)	Diff (ppm)	Diff (abs. ppm)	Diff (mDa)
	C15 H8 Cl2 O6	80.55	353.96743		353.96979	6.67	6.67	2.36

Species	Ion Formula	m/z	Height	Score (MFG)	Score (MFG, MS)	Score (MFG, mass)	Score (MFG, abund)	Score (MFG, iso. spacing)
(M-H)-	C15 H7 Cl2 O6	352.9625	47535.6	80.55	80.55	70.32	98.15	79.87

m/z	m/z (Calc)	Diff (ppm)	Diff (mDa)	Height	Height (Calc)	Height %	Height % (Calc)	Height Sum %	Height Sum% (Calc)
352.9604	352.9625	6.1	2.2	47535.6	48604.2	100	100	47	48.1
353.9615	353.9659	12.35	4.4	7990.3	8035.6	16.8	16.5	7.9	8
354.957	354.9599	8.24	2.9	33316	32323.5	70.1	66.5	33	32
355.9606	355.9631	7.11	2.5	5261.8	5270.8	11.1	10.8	5.2	5.2
356.9547	356.9578	8.57	3.1	5676.6	5768.7	11.9	11.9	5.6	5.7
357.9533	357.9607	20.85	7.5	874	905.9	1.8	1.9	0.9	0.9

Figure S40. HRESIMS spectrum of compound **6**

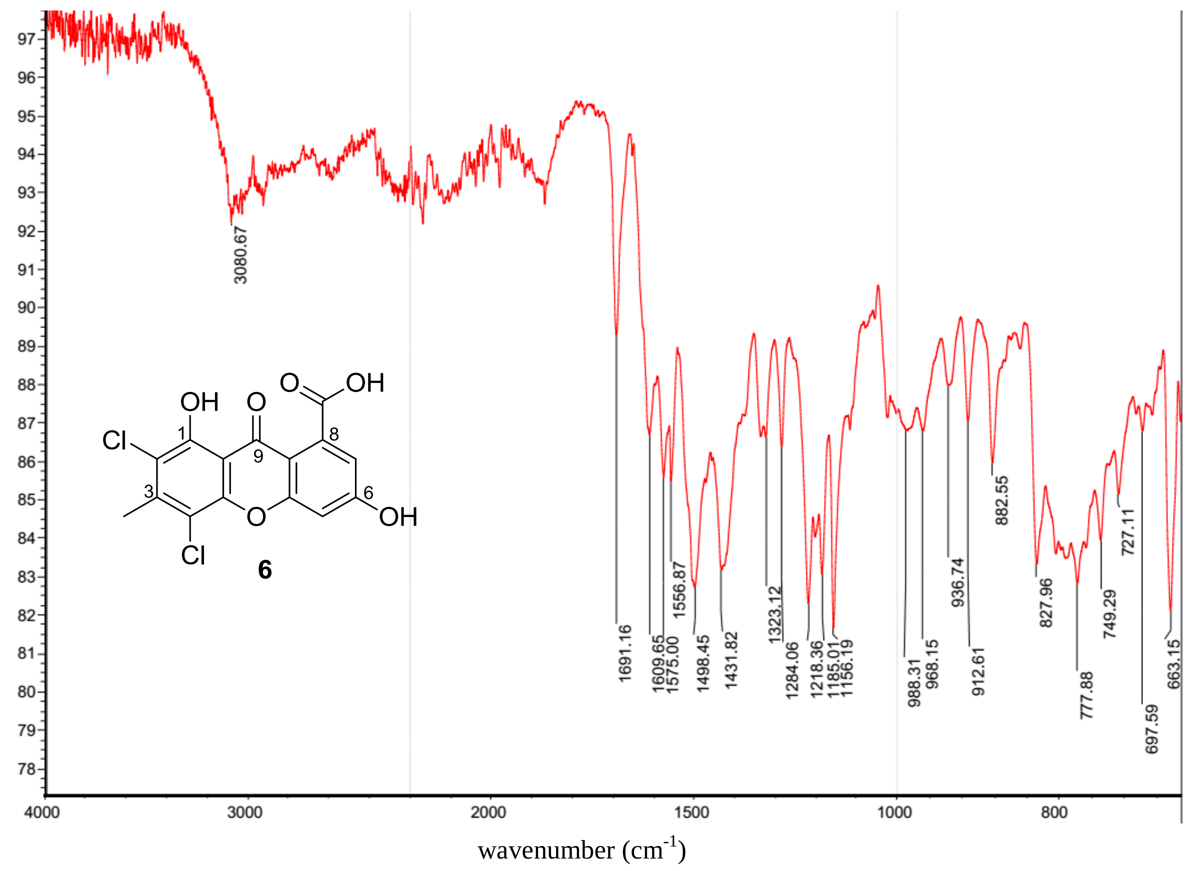

Figure S41. IR spectrum of compound **6**

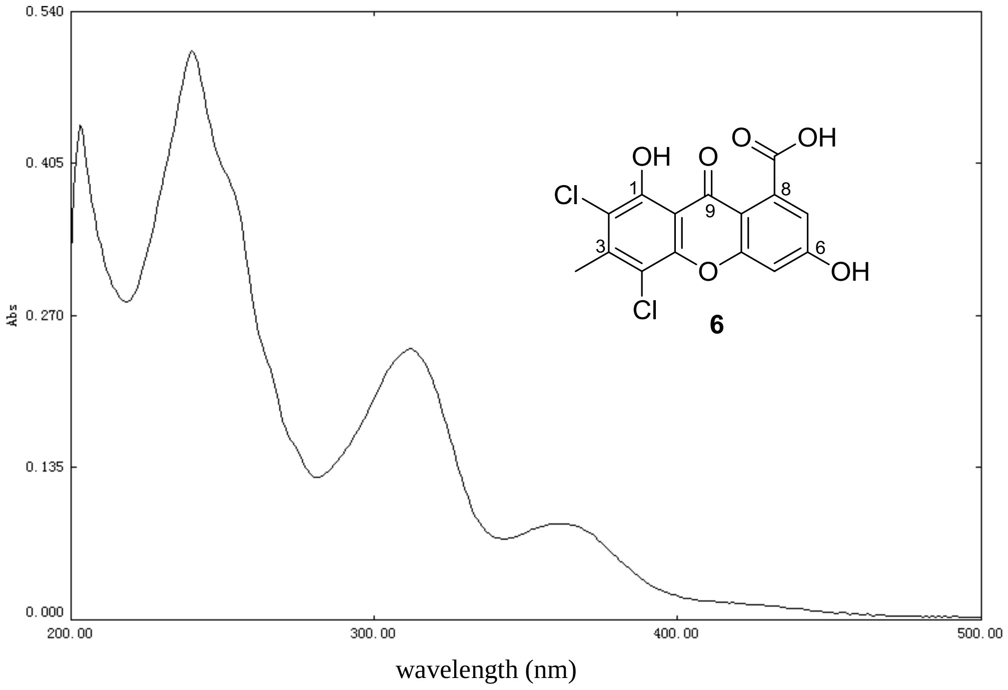

Figure S42. UV spectrum of compound **6** in MeOH
